# Single‐cell multi‐omics analysis presents the landscape of peripheral blood T‐cell subsets in human chronic prostatitis/chronic pelvic pain syndrome

**DOI:** 10.1111/jcmm.16021

**Published:** 2020-10-30

**Authors:** Meng Zhang, Yi Liu, Junyi Chen, Lei Chen, Jialin Meng, Cheng Yang, Shuiping Yin, Xiansheng Zhang, Li Zhang, Zongyao Hao, Xianguo Chen, Chaozhao Liang

**Affiliations:** ^1^ Department of Urology The First Affiliated Hospital of Anhui Medical University Hefei China; ^2^ Institute of Urology Anhui Medical University Hefei China; ^3^ Anhui Province Key Laboratory of Genitourinary Diseases Anhui Medical University Hefei China; ^4^ Institute of Urology of Shenzhen University The Third Affiliated Hospital of Shenzhen University Shenzhen Luohu Hospital Group Shenzhen China

**Keywords:** chronic prostatitis/chronic pelvic pain syndrome, effector T cell, single‐cell multi‐omics, T regular cell

## Abstract

Cumulative evidence suggests that abnormal differentiation of T lymphocytes influences the pathogenesis of chronic prostatitis/chronic pelvic pain syndrome (CP/CPPS). Thus, understanding the immune activation landscape of CP/CPPS would be helpful for improving therapeutic strategies. Here, we utilized BD™ AbSeq to digitally quantify both the protein and mRNA expression levels in single peripheral blood T cells from two CP/CPPS patients and two healthy controls. We utilized an integrated strategy based on canonical correlation analysis of 10 000+ AbSeq profiles and identified fifteen unique T‐cell subpopulations. Notably, we found that the proportion of cluster 0 in the CP/CPPS group (30.35%) was significantly increased compared with the proportion in the healthy control group (9.38%); cluster 0 was defined as effector T cells based on differentially expressed genes/proteins. Flow cytometry assays confirmed that the proportions of effector T‐cell subpopulations, particularly central memory T cells, T helper (Th)1, Th17 and Th22 cells, in the peripheral blood mononuclear cell populations of patients with CP/CPPS were significantly increased compared with those of healthy controls (*P* < 0.05), further confirming that aberration of effector T cells possibly leads to or intensifies CP/CPPS. Our results provide novel insights into the underlying mechanisms of CP/CPPS, which will be beneficial for its treatment.

## 1 INTRODUCTION

Chronic pelvic pain syndrome (CPPS) is characterized by pain perceived in structures related to the pelvis for at least six months without proven infection or other obvious local pathology,^1^ and it is highly prevalent, affects millions of people worldwide and impairs the quality of life in a manner similar to that observed with congestive heart failure, Crohn's disease, diabetes mellitus or angina.^2^ Although multimodal therapies seem to be the most successful,^3^ CPPS remains a major challenge and is a source of frustration for both patients and physicians, as available treatments often fail and a 'panacea' is still lacking. Currently, the lack of a recognized model is one of the most urgent problems in the mechanistic study of prostatitis.

The most common type of prostatitis is category III, also known as chronic prostatitis (CP)/CPPS. Diverse aetiologies regarding the pathogenesis of CP/CPPS have been proposed, which suggests that immune, neurological, endocrine and psychological factors may be involved. Of the suggested theories on the aetiology of CP/CPPS, the autoimmune basis is dominant. In addition, animal models have also been used to test autoimmunity. In rats, experimental autoimmune prostatitis could be induced by immunization with a male accessory gland blend, which is a mixed organ homogenate of the prostate (ventral, dorsal and lateral), and the coagulating gland.^4^ This induction is characterized by specific cell‐mediated responses and the infiltration of CD4+ and CD8+ T cells.^5^ The T cells sensitized by the male accessory gland are able to expand and differentiate by themselves in response to homogenates of the prostate.^6^ Lymphocytic infiltration of the stroma and periglandular region of the dorsal and lateral lobes is induced after immunization with only the rat ventral prostate.^7^ Notably, parallel findings are observed among humans, indicating that a similar process occurs.

A study found that seminal antigens generated from normal asymptomatic men promote the proliferation of T cells that have been extracted from patients with CP/CPPS, although this phenomenon is not seen in healthy control T cells.^8^ A subsequent study found that the prostate‐specific antigen (PSA) in 5/14 patients increased T‐cell proliferation, whereas there was no response to prostatic acid phosphatase.^9^ These studies suggest an autoimmune basis for the aetiology of CP/CPPS. Interestingly, approximately 9% of spermatozoa are described as intraprostatic upon autopsy, and they mainly exist in the peripheral zone.^10^ Thus, as these non‐prostatic substances, such as spermatozoa, exist in the prostate, there is support for the hypothesis that the antigens leading to autoimmune response may not only be of prostatic origin. Testing the T‐cell status underlying the pathogenesis of CP/CPPS is beneficial for identifying the pathogenic antigens.

Increasing evidence suggests that the abnormal differentiation of T cells influences the pathogenesis of CP/CPPS. Understanding the immune activation landscape of CP/CPPS is helpful for developing novel therapeutic strategies.^11^, ^12^, ^13^ Single‐cell multi‐omics (protein and mRNA) sequencing is a powerful tool capable of producing a complete catalog of cell types and states present within a sample. Here, we employed BD™ AbSeq on the Rhapsody™ platform in the analysis of peripheral blood T cells from patients with CP/CPPS and healthy individuals. Our findings delineate the complex array of T‐cell status in human blood and suggest that effector T cells, particularly central memory T cells, T helper (Th)1, Th17, and Th22 cells, as well as Treg cells, might be the causes of CP/CPPS.

## 2 MATERIALS AND METHODS

### 2.1 Patient recruitment

The present study was approved by the ethics committee of the First Affiliated Hospital of Anhui Medical University. Patients were recruited from the outpatient department, and controls were healthy donors. Informed consent was obtained from the participants before experiments were performed. Two patients with CP/CPPS and two healthy adults have included in single‐cell multi‐omics sequencing.

### 2.2 Sample preparation

**FIGURE 1:**
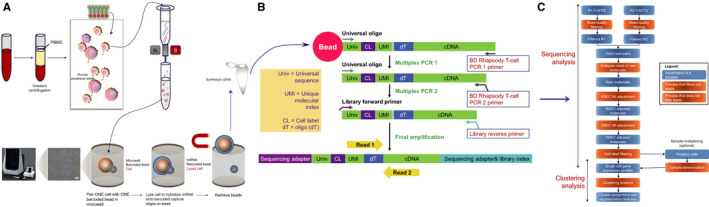
Single‐cell isolation, library preparation and bioinformatic analyses. A, The CD3+ T‐cell enrichment by CD3+ T‐cell enrichment kit, followed by single‐cell isolation and library construction; B, the flow showed reverse transcription and library construction details; and C. bioinformatic analyses procedure

Human peripheral blood samples were centrifuged in a Ficoll gradient to isolate peripheral blood mononuclear cells (PBMCs) (Figure 1A). Then, to obtain a high‐resolution identification of the T‐cell subsets, PBMCs were subjected to magnetic‐activated cell sorting (MACS) with human CD3 MicroBeads (130‐050‐101, Miltenyi Biotec, Bergisch Gladbach, Germany) according to the manufacturer's instructions. Cell viability was evaluated by Trypan blue staining. Then, the cells were washed and resuspended in PBS for BD Rhapsody™ System multi‐omics sequencing.

### 2.3 Single‐cell multi‐omics sequencing

BD Rhapsody™ T cell Panels and BD^®^ AbSeq Assays were used to map T‐cell immune composition and identify CP/CPPS‐related functional T‐cell subsets. The T‐cell sequence panel included 260 genes related to immune responses mediated by various kinds of T cells. The BD AbSeq panel contained 15 proteins, which are acknowledged surface markers used to characterize T‐cell subpopulations and functional states. The BD AbSeq panel was conducted first. Briefly, oligo‐conjugated antibodies (633781, Human Single‐Cell Multiplexing Kit, BD Bioscience, New Jersey, USA) were diluted with BD Stain Buffer (Cat. No. 554656, BD Bioscience, New Jersey, USA) to a volume of 110 μL. The freshly prepared single‐cell suspension was centrifuged and resuspended in 110 μL of BD Stain Buffer. Then, 100 μL of single‐cell suspension and antibody solution was mixed and incubated for 45 minutes on ice. Then, the labelled samples were washed with 2 mL of BD Stain Buffer for subsequent RNA extraction.

Single‐cell capture was achieved by a random distribution of a single‐cell suspension across over 200 000 microwells through a limited dilution approach. Beads with oligonucleotide barcodes were added to saturation to ensure that a bead was paired with a cell in a microwell. Cell lysis buffer was used to release polyadenylated RNA molecules and hybridize RNA to the beads. Then, beads were harvested into a single tube for reverse transcription. Upon cDNA synthesis, each cDNA molecule was tagged on the 5’ end with a molecular index and a label indicating its cell of origin. Libraries were prepared using the single‐cell amplification workflow, which is specifically demonstrated in Figure 1B. Sequencing was performed with Illumina NovaSeq according to the manufacturer's instructions (Illumina, Inc San Diego, CA, USA).

### 2.4 Data analysis

Read quality was qualified and filtered based on read length (R1 <66 or R2 <64, the ratio of R1/R2), mean base quality score (read score <20) and single‐nucleotide frequency (SNF, R1_SNF ≥0.55, R2_SNF ≥0.80) (Figure 1C). Then, the quality‐filtered R1 reads were analysed to identify the cell label section sequence (CLS), common sequence (L), unique molecular identifier (UMI) sequence and poly(T) tail. Information on the cell label is captured by bases in three sections (CLS1, CLS2 and CLS3) along each R1 read. Two common sequences (L1 and L2) separate the three CLSs, and the presence of L1 and L2 indicates the way capture oligonucleotide probes on the beads are constructed. By design, each CLS has one of 96 pre‐defined sequences. Reads are first checked for perfect matches in all three pre‐designed CLS sequences at the expected locations, CLS1: position 1‐9, CLS2: position 22‐30, and CLS3: position 44‐52. Only reads with perfect matches were kept and subjected to another round of filtering to recover reads with base substitution, insertion and deletions caused by oligonucleotide synthesis. The UMI is a string of eight random bases immediately downstream of CLS3. The UMI sequence would be at position 53‐60 if the CLSs perfectly match or with base substitution. For CLSs with insertions and deletions, the UMI sequence is eight bases immediately following the end of the identified CLS3. After the UNI, a poly(T) tail, which is the polyadenylation poly(A) complement of an mRNA molecule, was expected. Each read with a valid cell label was kept for further consideration only if over 6 out of 8 bases after the UMI were found to be Ts.

Following R1 annotation, Bowtie 2 version 2.2.9 was used to map the filtered R2 reads to the reference panel sequence. The R2 read was considered valid if all the following criteria were matched: the read aligned uniquely to a transcript sequence in the reference; the R2 alignment began within the first five nucleotides; the length of the alignment that could be a match or mismatch in the Compact Idiosyncratic Gapped Alignment Report string was >60; and the read did not align to phiX174. Reads paired with a valid R1 and valid R2 reads were retained for further analyses. Valid R1 reads required identified CLSs, a UMI sequence with non‐N bases and poly (T) tail. Valid R2 reads needed to uniquely map to a gene in panel with the correct PCR2 primer sequence at the start and to have an alignment of over 60 bases in length.

Reads with the same cell label, same UMI sequence and the same gene were collapsed into a single raw read of a molecule. The number of reads related to each raw molecule ID was reported as the raw adjusted sequencing depth. To remove the single‐base substitution errors, which were identified and adjusted to the parent UMI barcode using recursive substitution error correction. Other UMI errors derived from library preparation steps or sequencing base deletions were adjusted using distribution‐based error correction.

To distinguish cell labels associated with putative cells from those associated with noise, a multistep algorithm was designed for filtering cell labels. The number of reads for each cell was plotted on a log_10_‐transformed cumulative curve, with cells sorted in descending order by the number of reads. In a typical experiment, a distinct inflection point was observed, as indicated by the red vertical line. The algorithm found the minimum derivative along the cumulative reads curve as the inflection point. Cell labels to the left of the minimum second derivative were most likely derived from a cell capture event and were considered to be signals. The remaining cell labels to the right of the minimum second derivative were considered noise.

The R package Seurat was used to analyse the matrix obtained from the BD pipeline, and normalize the data, as well as reduce dimensionality and clustering, and identify differential expression. We used the Seurat alignment method canonical correlation analysis^14^ for integrated analysis of datasets. For clustering, highly variable genes were selected, and the principal components based on those genes were used to build a graph, which was segmented with a resolution of 0.6. Based on the filtered gene expression matrix produced by Seurat, sample differential expression analysis was carried out using the edgeR package to obtain zone‐specific marker genes.^15^, ^16^ In addition, KEGG analysis was performed based on an online database (https://david.ncifcrf.gov/ ).

### 2.5 Flow cytometry verification

Briefly, PBMCs were acquired by gradient centrifugation. Then, the cells were incubated with various fluorescein‐labelled antigens for surface staining (Table S1). PerCP/Cyanine5.5‐conjugated CD3 (300430, Biolegend, San Diego, CA, USA) and FITC‐conjugated CD4 (11‐0047‐41, eBioscience, San Diego, CA, USA) were used for Th1, Th2, Th9, Th17 and Th22 cells, and PE‐conjugated CD25 (557138, BD Bioscience, New Jersey, USA) and FITC‐conjugated CD4 were used to stain Treg cells. Then, samples stained for Th1, Th2, Th9, Th17 and Th22 cells were cultured with 1640 medium containing PMA (0.5 μm, HY‐18739, MedChemExpress, New Jersey, USA), ionomycin (1 μm, 70‐CS0002, MultiSciences, Zhejiang, China) and monensin (0.5 μm, 70‐CS0004, MultiSciences, Zhejiang, China) for four hours for cytokine stimulation and cell staining. Treg cells were directly moved on to the next step without stimulation. Then, all the samples were fixed and permeabilized with a cell fixation/permeabilization kit (00‐5523‐00, eBioscience, San Diego, CA, USA). For sample staining, Treg cells were incubated with eFluor 660‐conjugated FoxP3 (50‐4777‐42, eBioscience, San Diego, CA, USA); for sample staining, Th1 cells were incubated with PE‐conjugated IFN‐γ (559326, BD Bioscience, New Jersey, USA); for sample staining, Th2 cells were incubated with PE‐conjugated IL‐4 (12‐7049‐42, eBioscience, San Diego, CA, USA); for sample staining, Th9 cells were incubated with PE‐conjugated IL‐9 (12‐7098‐41, eBioscience, San Diego, CA, USA); for sample staining, Th17 cells were incubated with PE‐conjugated IL‐17A (560436, BD Bioscience, New Jersey, USA); and for sample staining, Th22 cells were incubated with PE‐conjugated IL‐17A and APC‐conjugated IL‐22 (17‐7222‐82, eBioscience, San Diego, CA, USA). Finally, samples were washed and sorted with a BD FACSCalibur system. The effector memory T cell and central memory T cells were sorted by using the PerCP/Cyanine5.5‐conjugated CD3 (300430, Biolegend, San Diego, CA, USA), FITC‐conjugated CD4 (11‐0047‐41, eBioscience, San Diego, CA, USA), PE‐conjugated CD45RA (304108, Biolegend, San Diego, CA, USA) and APC‐conjugated CD62L (559772, BD Pharmingen, San Diego, CA, USA).^17^ The flow cytometry results were analysed with FlowJo 10.0.7 software (Flowjo, Ashland, OR, USA).

## 3 RESULTS

### 3.1 Sample characteristics and single T‐cell profile generation

We collected PBMCs from two CP/CPPS patients and two healthy controls (Table S2). The flow chart shows the detailed steps of single‐cell RNA sequencing, which is described in Figure 1. Then, a CD3+ T‐cell enrichment kit was employed to enrich the CD3+ T cells from PBMCs. After testing the viability of the cells with Trypan blue staining (Table S3), the live cells were used to perform library construction and simultaneous mRNA and protein quantification at the single‐cell level by the BD™ AbSeq on the Rhapsody™ platform.

### 3.2 Quality control

Read quality was qualified and filtered based on read length (R1 <66 or R2 <64), mean base quality score (read score <20) and SNF (R1_SNF ≥0.55, R2_SNF ≥0.80). Reads with the same cell label, same UMI sequence and the same gene were collapsed into a single raw read of a molecule. We analysed the correlation between cell and gene/UMI counts in both the healthy control and prostatitis subgroups, and according to which, the cells in low quality (determined by detected UMI or gene counts) were removed from subsequent analyses (Table S4, and Figure S1). In addition, the expression landscape of fifteen proteins among all cells is described in Figure S2. The number of reads related to each raw molecule ID was reported as the raw adjusted sequencing depth. Single‐base substitution errors, which were identified and adjusted to the parent UMI barcode using recursive substitution error correction, were removed. Other UMI errors derived from library preparation steps or sequencing base deletions were later adjusted using distribution‐based error correction.

To distinguish cell labels associated with putative cells from those associated with noise, a multistep algorithm was designed for filtering cell labels. The number of reads from each cell was plotted on a log_10_‐transformed cumulative curve, with cells sorted in descending order by the number of reads (Figure S3). The R package Seurat was used to analysing the matrix obtained from BD pipeline and to normalize data; in addition, this package was used for dimensionality reduction, clustering and determination of differential expression.^14^ After removing the low‐quality cells, the remaining 10 000+ AbSeq profiles (merged from two cases and two controls) were used for subsequent analysis.

### 3.3 Single‐cell RNA‐seq analysis reveals distinct subsets of cell

For clustering, highly variable genes were selected, and the principal components based on those genes and proteins were used to build a graph, which was segmented with a resolution of 0.6. Based on the filtered gene expression matrix produced by Seurat, sample differential expression analysis was carried out using the edge T package to obtain zone‐specific marker genes and proteins (Table S5).

**FIGURE 2:**
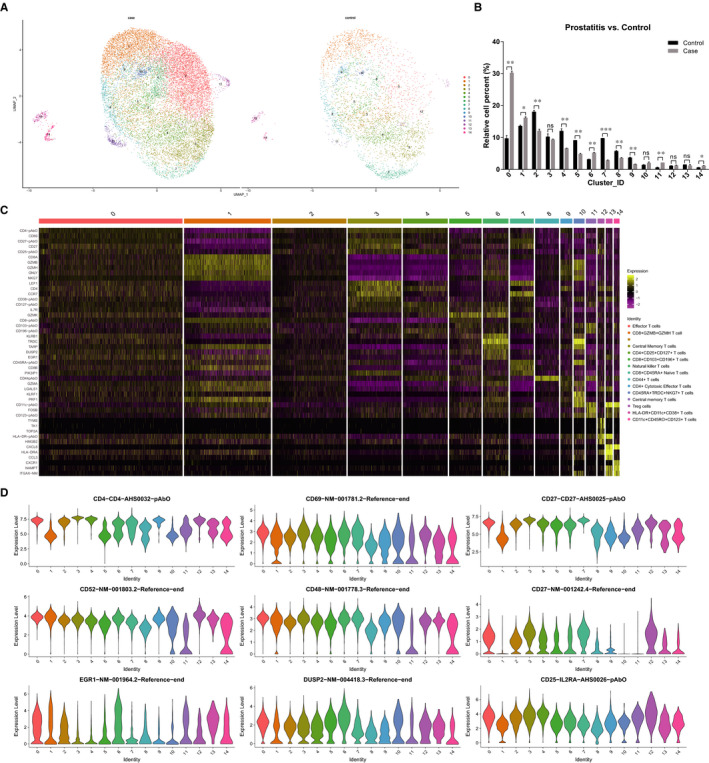
Single‐cell multi‐omics analysis revealed 15 distinct T‐cell subsets. A, UMAP plot showing dimensional reduction of the distribution of 10 000+ individual T cells extracted from two CP/CPPS cases and two healthy controls; B, the proportion variations of the T‐cell subsets between CP/CPPS cases and healthy controls (*P < 0.05; **P < 0.01); C, heatmap plot showing clustering with top 5 highly expressed genes/proteins within each transgenic line. Representative genes/proteins found within each specific cell lineage are denoted to the left. The cells were defined based on the marker genes/proteins identified and labelled on the right of the plot. D, Violin plots comparing the expression of key differentially expressed genes/proteins in different clusters

**FIGURE 3:**
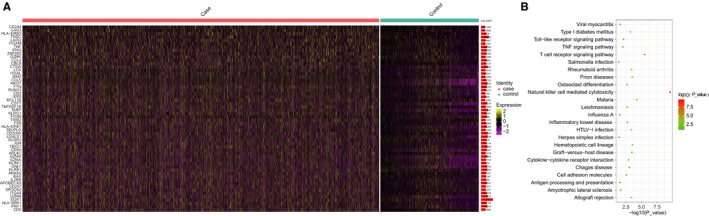
Analyses of the differentially expressed genes/proteins between CP/CPPS cases and healthy controls and their corresponded pathways. A, The differentially expressed genes between CP/CPPS cases and healthy controls; B, the pathway enrichment of the differentially expressed genes/proteins between CP/CPPS cases and healthy controls. CP/CPPS, chronic prostatitis/chronic pelvic pain syndrome

To reveal the intrinsic structure and potential functional subsets of the overall T‐cell populations, we performed unsupervised clustering of CD3+ T cells from PBMCs. A total of fifteen stable clusters emerged based on 10 000+ AbSeq profiles, and each had unique signature genes (Figure 2A). Notably, we analysed the CD3+ T‐cell proportion variations between the CP/CPPS group and healthy controls, and the results suggested that the proportion of cluster 11 in the CP/CPPS group (2.11%) was significantly increased (by 3.4‐fold) compared with the proportion in the healthy control group (0.62%); this result was followed by the proportion of cluster 0 in the CP/CPPS group (30.35%), which was significantly increased (by 3.2‐fold) compared with the proportion in the healthy control group (9.38%) (Figure 2B). We analysed the differentially expressed genes (DEGs) and surface proteins in each cluster, according to which functional subsets of CD3+ T cells were defined (Figure 2C). In addition to cluster 2, the functional subsets from cluster 0 to cluster 14 were defined as based on the differentially expressed genes or proteins of each cluster in order as follows: effector T cells, CD8 + GZMB + GZMH T cells, central memory T cells, CD4 + CD25 + CD127+ T cells, CD8 + CD103 + CD196+ T cells, natural killer T cells, CD8 + CD45RA+ naive T cells, CD44+ T cells, CD4 + cytotoxic effector T cells, CD45RA + TRDC + NKG7+ T cells, central memory T cells, Treg cells, HLA‐DR + CD11c+CD38+ T cells, and CD11c + CD45RO+CD123+ T cells (Figure 2D and Figure S4). Further, we compared the gene expression differences between cells derived from prostatitis patients and healthy controls in each cluster and performed pathway enrichment analyses to identify critical pathways (Figure [Link], [Link], [Link], and Table S6). These results would be helpful for future basic research to annotate the biological function of these critical candidates in regulating specific cell subsets during the pathogenesis of CP/CPPS. In addition, we also compared the DEGs between overall cells derived from CP/CPPS and healthy control subjects and found that these genes were mostly enriched in natural killer cell‐mediated cytotoxicity, Th1 and Th2 cell differentiation, hematopoietic cell lineage, allograft rejection, graft‐versus‐host disease, T‐cell receptor signalling pathway, *etc* (Table S7 and Figure 3). These results further demonstrated that under CP/CPPS conditions, T cells were activated.

**FIGURE 4:**
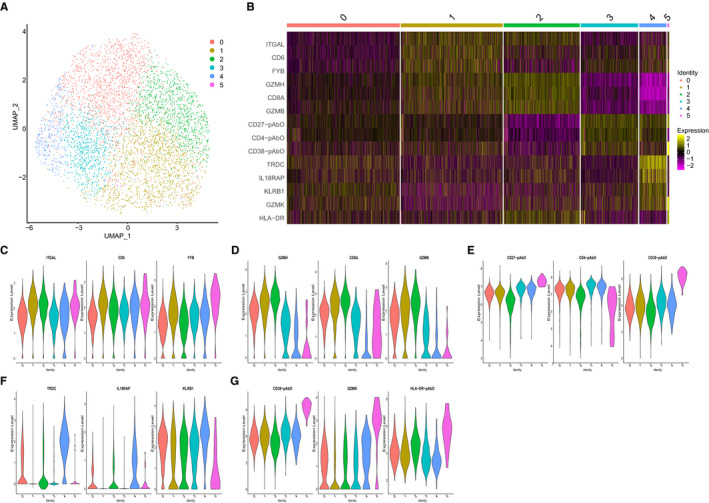
Secondary UMAP analysis of cluster 0. A, UMAP analysis and cluster allocation for the single cells in cluster 0; B, heatmap plot showed the marker genes/proteins in each newly defined cluster; and C‐G. the identified marker genes/proteins in cluster 0 to 5. UMAP, Uniform Manifold Approximation and Projection

With limited information provided by the marker genes or proteins in cluster 0 (Figure 3D), it is difficult to define the subtypes of T cells. Furthermore, we performed principal component analysis on cluster 0. Based on the differentially expressed genes and proteins, cluster 0 was divided into six new subsets (Figure 4). However, we failed to define these new subsets according to the marker genes and proteins, but we compared the DEGs between cells derived from healthy controls and prostatitis patients in each cluster and performed the pathway enrichment analyses, which could help realize the underlying mechanisms of CP/CPPS in future research (Table S8 and Figures S8 and S9). Further, a flow cytometry assay is warranted to identify these specific T‐cell subsets.

### 3.4 Flow cytometry validation

**FIGURE 5:**
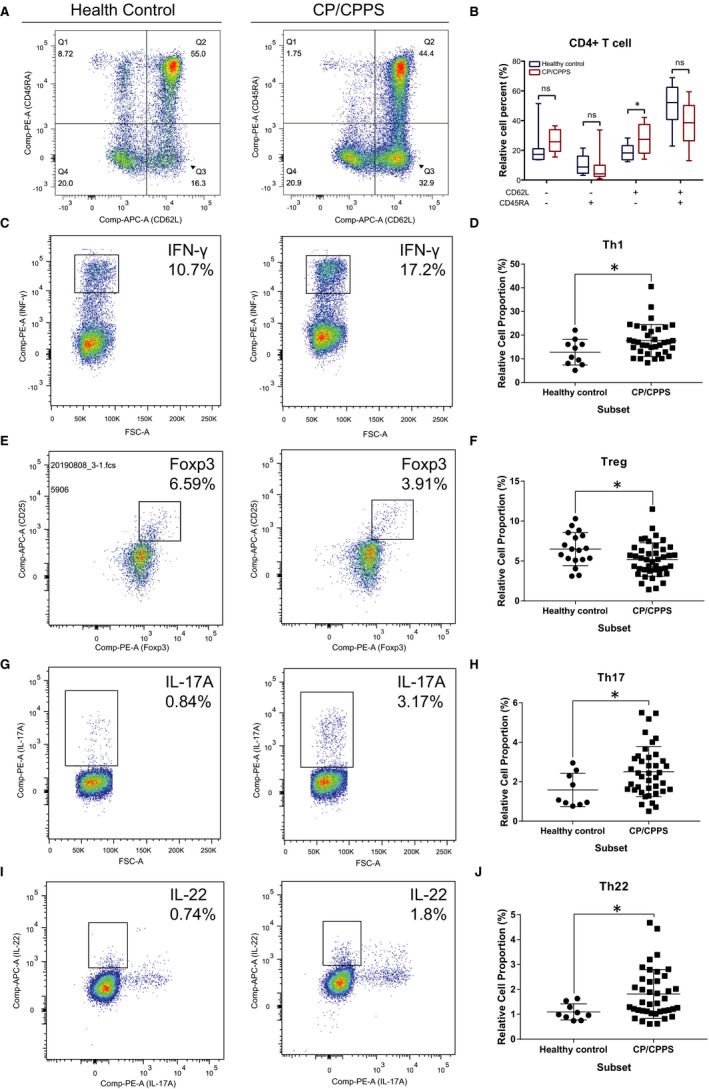
Flow cytometry revealed central memory T cell, Th1, Th17, Th22 and Treg proportions increased in PBMCs derived from CP/CPPS patients than healthy controls. PBMCs were incubated with various fluorescein‐labelled antigens for surface staining. PerCP/Cyanine5.5‐conjugated CD3 and FITC‐conjugated CD4 were used for the central memory T cell, Th1, Th17 and Th22 cell staining, and PE‐conjugated CD25 and FITC‐conjugated CD4 were incubated for staining Treg cells. After fixing and permeabilizing with cell fixation/permeabilization kit, (A) for samples staining central memory T cells were incubated with PE‐conjugated CD45RA and APC‐conjugated CD62L; (C) for samples staining, Th1 cells were incubated with PE‐conjugated IFN‐γ; (E) for samples staining, Treg cells were incubated with eFluor 660‐conjugated FoxP3; (G) for samples staining, Th17 cells were incubated with PE‐conjugated IL‐17A; and (I) for samples staining, Th22 cells were incubated with PE‐conjugated IL‐17A and APC‐conjugated IL‐22. The quantification data were presented in B, D, F, H and J. *P < 0.05; PBMC, peripheral blood mononuclear cell

Furthermore, because of the significance of effector T cells, we employed flow cytometry to validate these findings. We tested changes in the proportions of effector memory T cell, central memory T cell, Th1, Th2, Th9, Th17 and Th22 cells in PBMCs from patients with CP/CPPS and from healthy controls (Figure 5 and Figure S10). Our results suggested that the number of cells in the central memory T cell, Th1, Th17 and Th22 cell subsets in CP/CPPS cases was significantly increased compared with those in healthy controls (cases vs controls, central memory T cell: 27.45 ± 3.081 vs 18.97 ± 1.676, *P*‐value < 0.05; Th1: 17.75 ± 1.146 vs 12.81 ± 1.717, *P*‐value < 0.05; Th17: 2.506 ± 0.2009 vs 1.58 ± 0.2829, *P*‐value < 0.05; Th22: 1.861 ± 0.1262 vs 1.263 ± 0.3152, *P*‐value < 0.05; Figure 5). Furthermore, we also compared the variation in Treg cells in PBMCs derived from CP/CPPS patients and healthy controls and found that the proportion of Treg cells in CP/CPPS patients was significantly reduced compared with the proportion in healthy controls (Treg, case vs control: 5.16 ± 0.3192 vs 6.567 ± 0.5186, *P*‐value < 0.05; Figure 5); this result was consistent with the findings of our previous publications.^12^ In summary, our results further prove the hypothesis that abnormalities of the immune system may be one of the important factors leading to CP/CPPS.

## 4 DISCUSSION

Immune disorders and autoimmunity activation have been hypothesized to contribute to the development of CP/CPPS.^18^ The establishment and maintenance of immune responses, homoeostasis and memory depend on T cells, and the unbalance of T‐cell subsets are implicated as major drivers of many inflammatory and autoimmune diseases,^19^ while intervening the cell imbalance using the cytokines, gene therapy, vaccine and incomplete Freund's adjuvant are regarded as promising therapeutic options for these diseases. Recently, T cells had been proved to be related to chronic prostatitis progression. One study found reduced CD3+ and CD45+ cell infiltration in the prostate stroma after effective therapies.^20^ The other researchers also found that CD4+ T cells and macrophages are key factors in the development of CP/CPPS with CP rodent models.^21^ A recent study also found that CP/CPPS patients show Th1 and Th17 immune responses specific to prostate antigen associated with chronic inflammation of the male genital tract, which may underlie the induction and development of chronic pelvic pain.^22^ Further, Ruben D. Motrich et al’s study showed that IFN‐γ secreting Th1 lymphocytes are the driver cells of autoimmune prostatitis pathogenesis and chronic pelvic pain induction.^11^ Therefore, we hypothesized that the immune imbalance of T‐cell subpopulations may be associated with the progression of chronic prostatitis. However, the numbers, functions, cell types and gene expressions of T cells remain unknown in CP/CPPS. Herein, we analysed human CD3+ T cells isolated from the PBMCs of patients with CP/CPPS and healthy donors using BD™ AbSeq on the Rhapsody™ platform. We reported a number of key findings, including (a) the identification of the presence of substantial CD3+ T‐cell heterogeneity, including fifteen different T‐cell subsets (cluster 0 to cluster 14); (b) the identification of seven T‐cell subsets that were increased in patients with CP/CPPS and were determined to be predominantly in cluster 0 and cluster 11; (c) the determination that cluster 0 (defined as effector T cells) represented Th1, Th17 and Th22 cells; and (d) the finding that Treg cells, which can regulate effector T cells, were increasing in CP/CPPS patients. The most significant contributions of this study are the determination of a landscape of T‐cell distributions in patients with CP/CPPS and the discovery that the immune imbalance of central memory T cell, Th1, Th17, Th22 and Treg cells is the potential cause of CP/CPPS.

In our study, we found that the proportion of Th1 cells was increased in PBMCs obtained from CP/CPPS patients compared with healthy controls. IFN‐γ‐secreting Th1 cells are critical during EAP induction.^23^, ^24^, ^25^ In a recent study based on knocking out mice, Ruben D. Motrich et al^13^ found that the absence of Th1 or Th2 cytokines diminishes or enhances EAP susceptibility, respectively. The expression of Th1‐correlated chemokine receptors CXCR3 and CCR5 on these prostate‐specific effector T cells is associated with their homing to and infiltration of the prostate.^23^ Combined evidence provided by previous publications and our findings further supports the pathogenic role of Th1 in CP/CPPS. Further, we also observed that the proportion of central memory T cells were increased in CP/CPPS patients compared with healthy control. These clues support the role of immune abnormality in regulating the pathogenesis of CP/CPPS.

Treg cells are a T‐cell subset with immunosuppressive function. They regulate the immune response by secreting TGF‐β and IL‐10.^26^ Natural self‐tolerized Tregs, known as natural Tregs (nTregs) and inducible Tregs (iTregs), are the two major regulatory T‐cell subsets.^26^ nTreg cells generated by the thymus are CD4+ T cells, and they express CD25 and FoxP3; these cells account for 5%–10% of CD4+ T cells in the PBMCs and spleen tissues of both normal humans and mice. The majority of iTreg cells are FoxP3+ Treg cells, which are differentiated from traditional CD4+ T cells in the peripheral blood or are generated by antigenic stimulation in vitro. The mRNA level of the FOXP3 gene in Treg cells was significantly lower in CP/CPPS patients than it was in healthy controls; further, serum TGF‐β1 levels, rather than serum TNF‐α levels, were up‐regulated in CP/CPPS patients compared with healthy controls.^27^ Furthermore, Breser et al ^28^ found that compromising the function of Treg cells enhances the susceptibility of the CP/CPPS rat model. These results suggested that Treg cell function loss and tolerance might be critical mediators of inflammation. Notably, the research found that Treg cells suppress the autoimmune response of Th1/cytotoxic T‐cell 1 (Tc1) populations and reduce CXCR3 expression in effector T cells. The chemokine receptor CXCR3 is critical for the homing of effector T cells to the prostate,^25^ which played a role in the pathogenesis of the CP/CPPS mouse model. In our study, we found that the proportion of Treg cells was reduced in CP/CPPS patients compared with healthy controls, further supporting the hypothesis that Treg cells may participate in the pathogenesis of CP/CPPS.

Unlike the functional roles of Th1 and Treg cells, the functional role of Th22 cells in CP/CPPS has only been investigated in a few studies. Th22 cells are a new subset of T cells that are clearly separated from Th17 cells and other known T‐cell subsets with distinct gene expression profiles and functions.^29^ With the CCR6 + CCR4 + CCR10 + phenotype and aryl hydrocarbon receptor as the key transcription factor, Th22 cell subsets produce cytokines such as IL‐22, whose function depends on the activation of signal transduction and activators of transcription.^30^ IL‐22 is up‐regulated in patients with rheumatoid arthritis,^31^, ^32^ Crohn's disease,^33^, ^34^ psoriasis^35^, ^36^ and atopic dermatitis,^37^, ^38^ whereas it is down‐regulated in the serum of patients with sarcoidosis and systemic lupus erythematosus. Furthermore, it has been demonstrated that IL‐22 may be a promising potential therapeutic option for chronic inflammatory diseases, and treatment with recombinant cytokines or gene therapy delivery of IL‐22 may alleviate tissue destruction during inflammatory responses.^39^ Th22 cells play an important and complicated role in inflammatory and autoimmune diseases. Further functional studies are warranted to prove that Th22 cells have a role in CP/CPPS pathogenesis or progression. One limitation of the current study was when we performed the flow cytometry, we used the PMA to stimulate the T cells that could result in the endocytosis of CD4, causing potential bias. This kind of bias could be removed when the two groups used the same wayy.^40^, ^41^

Although we are just beginning to dissect the significance of central memory T cell, Th1, Th17, Th22, and Treg cells and the vast amount of single‐cell data uncovered by our study, we hope that our reported findings will impact the understanding of the mechanisms of CP/CPPS and that further manipulation of these cells will create better methods for restoring homoeostasis.

## CONFLICT OF INTEREST

All authors declared no competing interests.

## AUTHOR CONTRIBUTIONS

**Meng Zhang:** Conceptualization (equal); Funding acquisition (equal); Resources (equal); Supervision (equal). **Yi Liu:** Data curation (equal); Methodology (equal); Validation (equal). **Junyi Chen:** Validation (equal). **Lei Chen:** Validation (equal). **Jialin Meng:** Validation (equal). **Cheng Yang:** Data curation (equal); Funding acquisition (equal); Validation (equal). **Shuiping Yin:** Formal analysis (equal); Methodology (equal). **Xiansheng Zhang:** Resources (equal); Supervision (equal). **Li Zhang:** Resources (equal); Supervision (equal). **Zongyao Hao:** Resources (equal); Supervision (equal). **Xianguo Chen:** Resources (equal); Supervision (equal). **Chaozhao Liang:** Conceptualization (equal); Funding acquisition (equal); Supervision (equal).

## Supporting information

Fig S1

Fig S2

Fig S3

Fig S4

Fig S5

Fig S6

Fig S7

Fig S8

Fig S9

Fig S10

Table S1

Table S2

Table S3

Table S4

Table S5

Table S6

Table S7

Table S8

## Data Availability

The datasets used and/or analysed during the current study are available from the corresponding author on reasonable request.
