# Supplementary material for: Single‐cell multi‐omics analysis presents the landscape of peripheral blood T‐cell subsets in human chronic prostatitis/chronic pelvic pain syndrome
Source: J Cell Mol Med. 2020 Oct 30;24(23):14099–109. doi: 10.1111/jcmm.16021 (PMC7754003; doi:10.1111/jcmm.16021)
Supplement: Supplementary file 1 — Fig S1 [file JCMM-24-14099-s001.pdf]

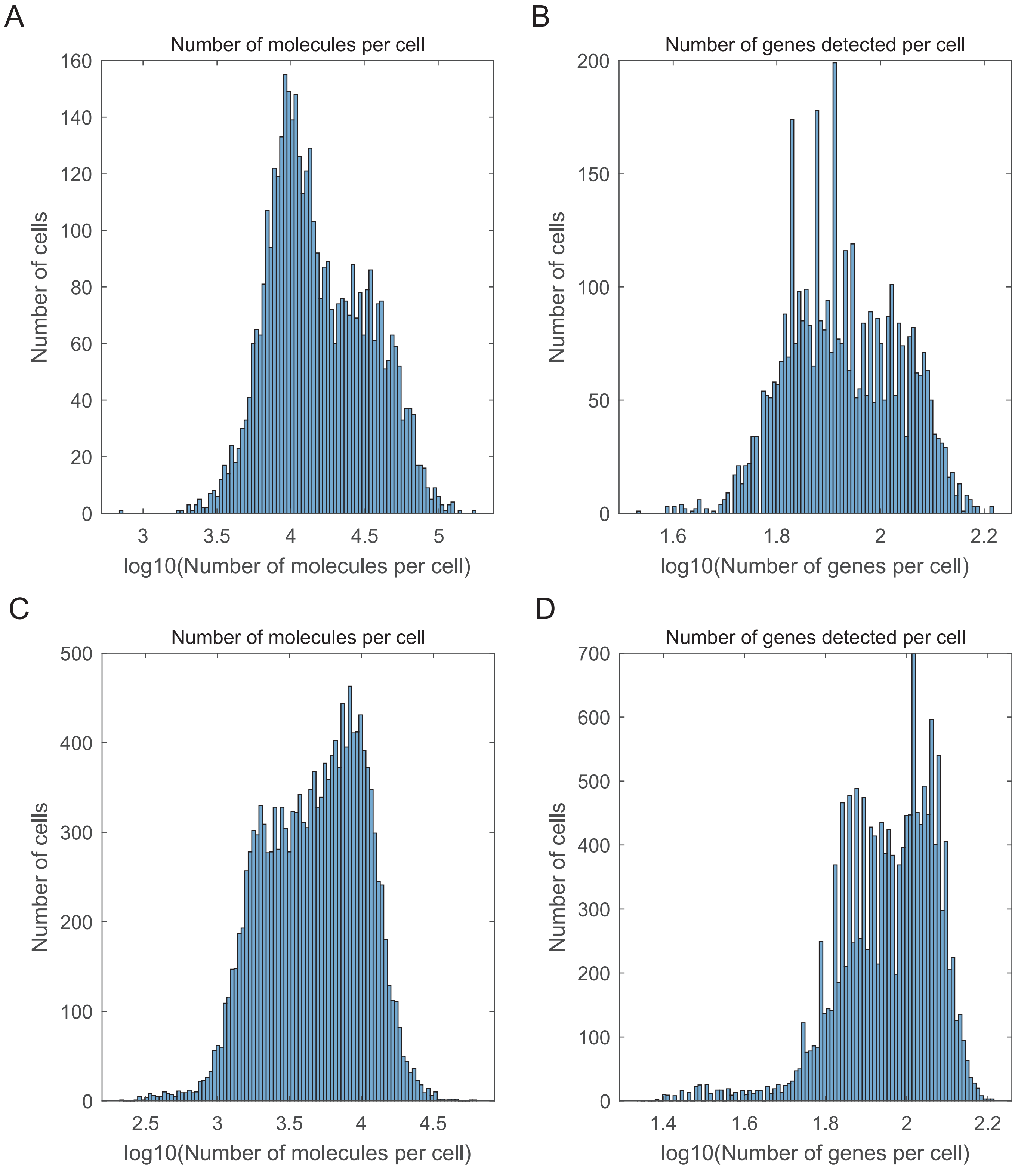

Supplementary figure 1. Quality control of the single-cell multi-omics data. A. Number of molecules per cell in the control samples; B. number of genes detected per cell in the control samples; C. number of cells with expression of gene in the chronic prostatitis samples; D. number of molecules detected from all cells in the chronic prostatitis samples.
