# Supplementary material for: Single‐cell multi‐omics analysis presents the landscape of peripheral blood T‐cell subsets in human chronic prostatitis/chronic pelvic pain syndrome
Source: J Cell Mol Med. 2020 Oct 30;24(23):14099–109. doi: 10.1111/jcmm.16021 (PMC7754003; doi:10.1111/jcmm.16021)
Supplement: Supplementary file 3 — Fig S3 [file JCMM-24-14099-s003.pdf]

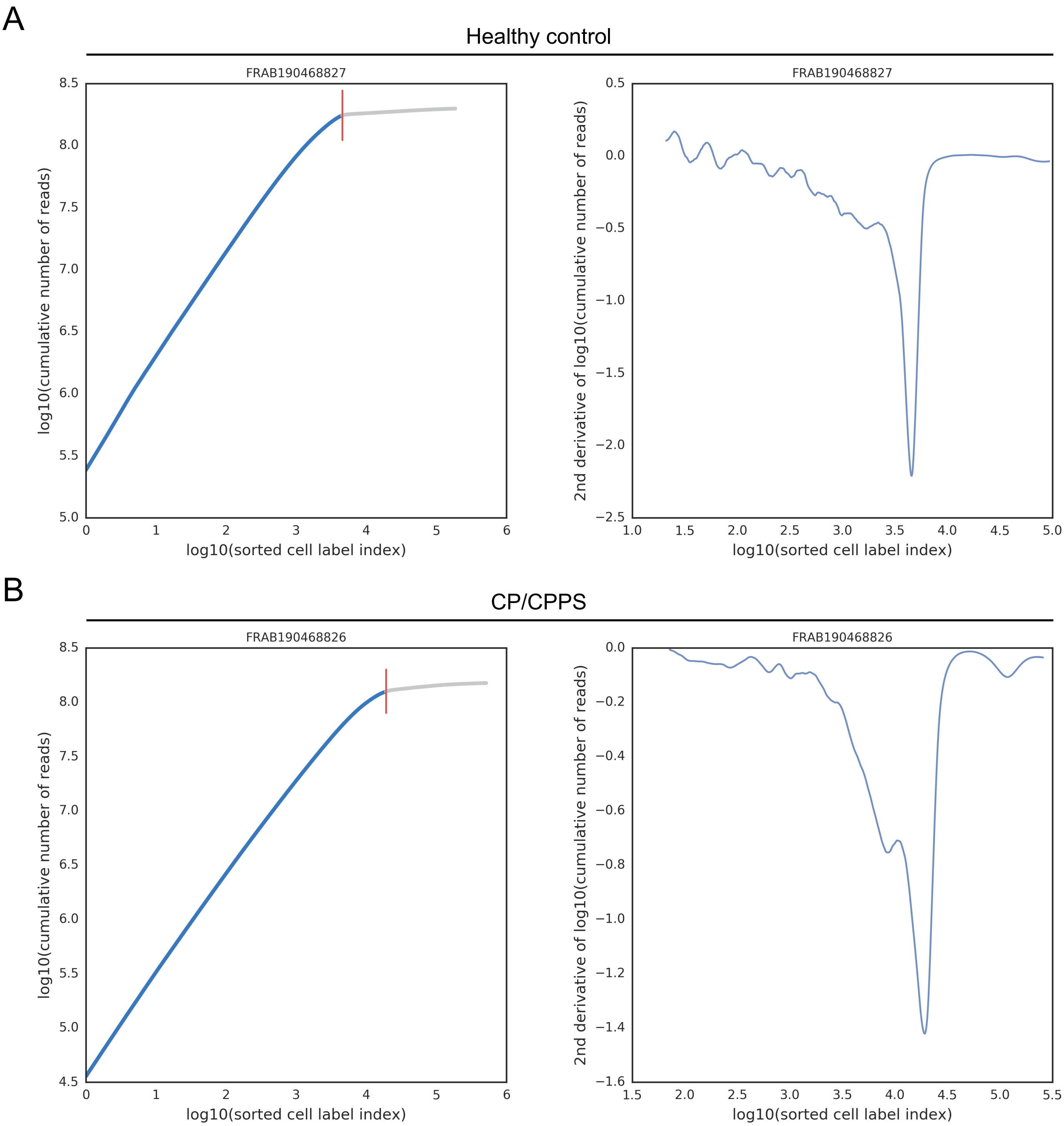

Supplementary figure 3. Determine putative cells in the control group (A) and case group (B). In theory, the cell labels obtained should be close to the number of captured cells. However, background noise might be introduced during the process to detect too many cells. To distinguish valid cells from the background noise, the BD pipeline identified cell numbers based on a second-order import algorithm. Relative to the background noise, the effective cells contained a high amount of mRNA, so the number of effective cells was more than the number of reads of the background noise. The number of Reads was very small; thus, an obvious inflection point would appear when accumulating to the background noise. The derivative of the accumulated value would obtain the derivative curve. The number of cells on the left of the derivative minimum (marked in red) was the number of valid cells, while the right was the background noise. CP/CPPS, chronic prostatitis/chronic pelvic pain syndrome.
