# Supplementary material for: Single‐cell multi‐omics analysis presents the landscape of peripheral blood T‐cell subsets in human chronic prostatitis/chronic pelvic pain syndrome
Source: J Cell Mol Med. 2020 Oct 30;24(23):14099–109. doi: 10.1111/jcmm.16021 (PMC7754003; doi:10.1111/jcmm.16021)
Supplement: Supplementary file 4 — Fig S4 [file JCMM-24-14099-s004.pdf]

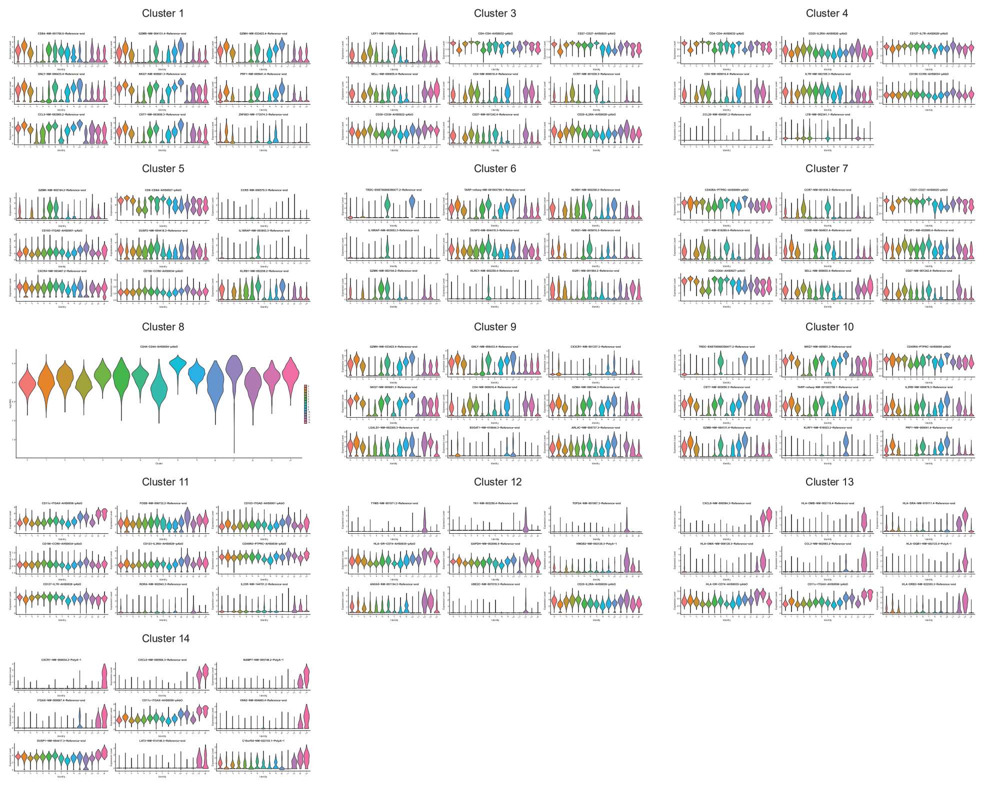

Supplementary figure 4. Expression of top 9 differentially expressed genes/proteins within each cluster. Note: no significant differentially expressed genes/proteins were identified in cluster 2.
