# Supplementary material for: Single‐cell multi‐omics analysis presents the landscape of peripheral blood T‐cell subsets in human chronic prostatitis/chronic pelvic pain syndrome
Source: J Cell Mol Med. 2020 Oct 30;24(23):14099–109. doi: 10.1111/jcmm.16021 (PMC7754003; doi:10.1111/jcmm.16021)
Supplement: Supplementary file 6 — Fig S6 [file JCMM-24-14099-s006.pdf]

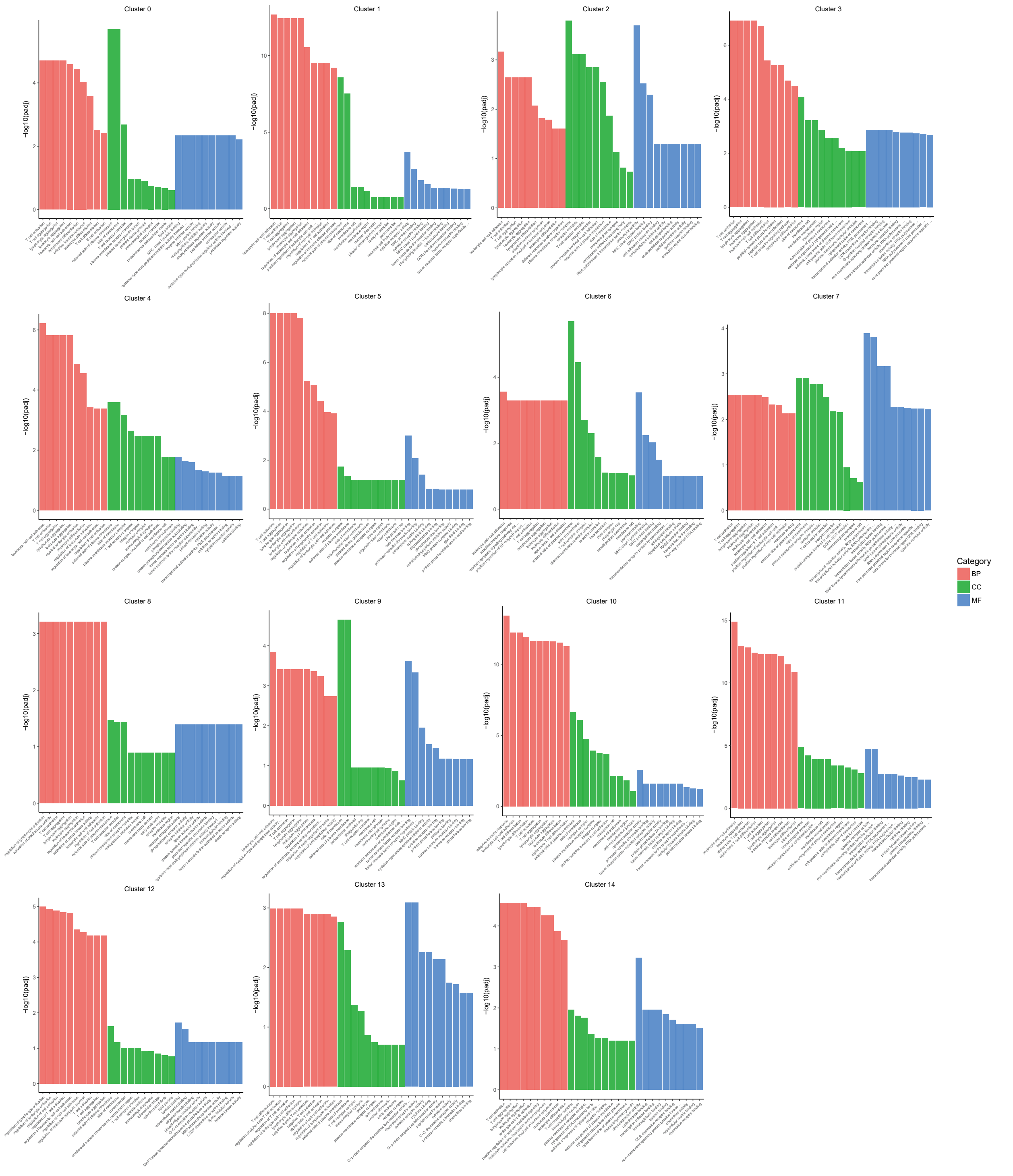

Supplementary figure 6. Gene ontology analyses for the differentially expressed genes between cells derived from prostatitis cases and healthy controls from cluster 0 and cluster 14. BP, biological process; CC, cellular component; MF, molecular function.
