# Supplementary material for: Single‐cell multi‐omics analysis presents the landscape of peripheral blood T‐cell subsets in human chronic prostatitis/chronic pelvic pain syndrome
Source: J Cell Mol Med. 2020 Oct 30;24(23):14099–109. doi: 10.1111/jcmm.16021 (PMC7754003; doi:10.1111/jcmm.16021)
Supplement: Supplementary file 8 — Fig S8 [file JCMM-24-14099-s008.pdf]

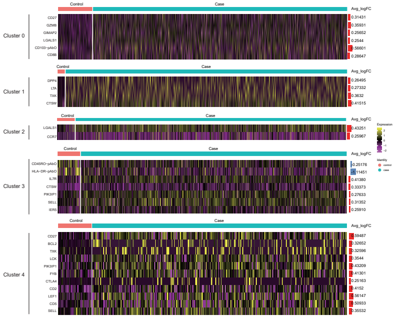

Supplementary figure 8. Differentially expressed genes/proteins between cells derived from prostatitis cases and healthy controls from cluster 0 to cluster 5. Note: no significant differentially expressed genes/proteins were identified in cluster 5.
