# Supplementary material for: Single‐cell multi‐omics analysis presents the landscape of peripheral blood T‐cell subsets in human chronic prostatitis/chronic pelvic pain syndrome
Source: J Cell Mol Med. 2020 Oct 30;24(23):14099–109. doi: 10.1111/jcmm.16021 (PMC7754003; doi:10.1111/jcmm.16021)
Supplement: Supplementary file 9 — Fig S9 [file JCMM-24-14099-s009.pdf]

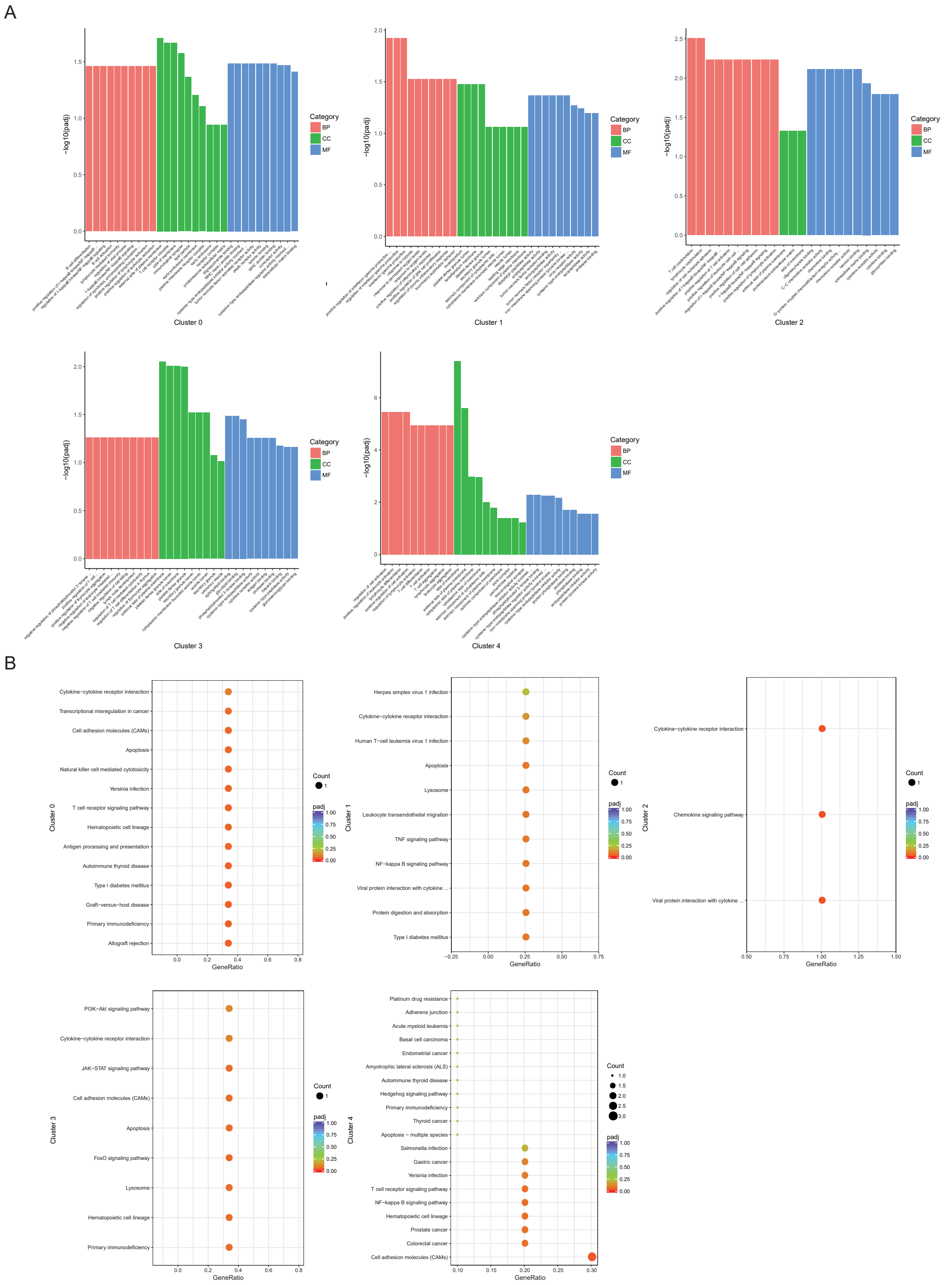

Supplementary figure 9. Gene ontology (A) and Kyoto Encyclopedia of Genes and Genomes (B) analyses for the differentially expressed genes between cells derived from prostatitis cases and healthy controls from cluster 0 and cluster 5. BP, biological process; CC, cellular component; MF, molecular function.
