# Supplementary material for: Single‐cell multi‐omics analysis presents the landscape of peripheral blood T‐cell subsets in human chronic prostatitis/chronic pelvic pain syndrome
Source: J Cell Mol Med. 2020 Oct 30;24(23):14099–109. doi: 10.1111/jcmm.16021 (PMC7754003; doi:10.1111/jcmm.16021)
Supplement: Supplementary file 10 — Fig S10 [file JCMM-24-14099-s010.pdf]

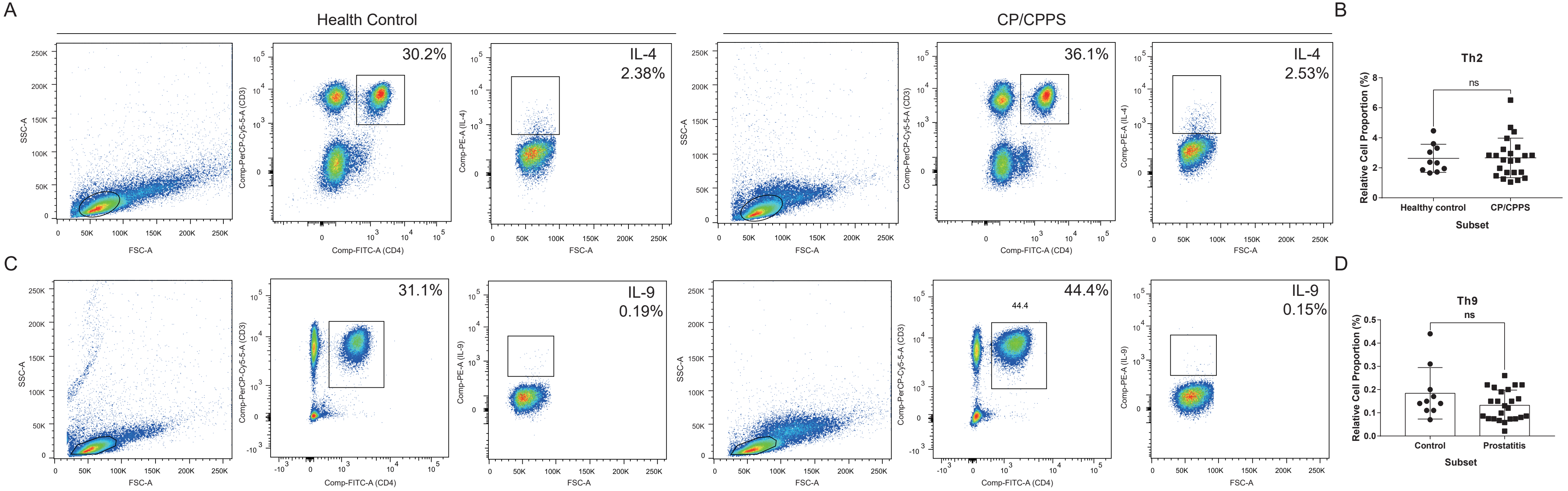

Supplementary figure 10. No significant difference was revealed by flow cytometry in the distributions of Th2 and Th9 cells in PBMCs derived from CP/CPPS patients and healthy controls. PBMC were incubated with various fluorescein-labeled antigens for surface staining. PerCP/Cyanine5.5 conjugated CD3 and FITC conjugated CD4 for the Th2 and Th9 cells. After fixing and permeabilizing with cell fixation/permeabilization kit, (A) for samples staining Th2 cells were incubated with PE-conjugated IL-4; (C) for samples staining Th9 cells were incubated with PE-conjugated IL-9. The quantification data were presented in B, and D. PBMC, peripheral blood mononuclear cell; CP/CPPS, chronic prostatitis/chronic pelvic pain syndrome.
