# Supplementary material for: Single‐cell multi‐omics analysis presents the landscape of peripheral blood T‐cell subsets in human chronic prostatitis/chronic pelvic pain syndrome
Source: J Cell Mol Med. 2020 Oct 30;24(23):14099–109. doi: 10.1111/jcmm.16021 (PMC7754003; doi:10.1111/jcmm.16021)
Supplement: Supplementary file 11 — Table S1 [file JCMM-24-14099-s011.docx]

**Supplementary table 1.** The main reagents and antibodies used in current study.

| **Reagents** | **Category number** | **Company** |
| --- | --- | --- |
| Human CD3 MicroBeads | 130-050-101 | Miltenyi Biotec |
| Human Single-Cell Multiplexing Kit | 633781 | BD Bioscience |
| BD Stain Buffer | 554656 | BD Bioscience |
| PerCP/Cyanine5.5 conjugated CD3 | 300430 | Biolegend |
| Anti-human FITC conjugated CD4 | 11-0047-41 | Ebioscience |
| Anti-human PE conjugated CD25 | 557138 | BD Bioscience |
| Anti-human eFluor 660 conjugated Foxp3 | 50-4777-42 | Ebioscience |
| Anti-human PE conjugated IFN-γ | 559326 | BD Bioscience |
| Anti-human PE conjugated IL-4 | 12-7049-42 | Ebioscience |
| Anti-human PE conjugated IL-9 | 12-7098-41 | Ebioscience |
| Anti-human PE conjugated CD45RA | 304108 | Biolegend |
| Anti-human APC conjugated CD62L | 559772 | BD Pharmingen |
| Anti-human PE conjugated IL-17A | 560436 | BD Bioscience |
| Anti-human APC conjugated IL-22 | 17-7222-82 | Ebioscience |
| Lonomycin | 70-CS0002 | MultiSciences |
| Monensin | 70-CS0004 | MultiSciences |
| Cell fixation/permeabilization kit | 00-5523-00 | Ebioscience |
