# Supplementary material for: Single‐cell multi‐omics analysis presents the landscape of peripheral blood T‐cell subsets in human chronic prostatitis/chronic pelvic pain syndrome
Source: J Cell Mol Med. 2020 Oct 30;24(23):14099–109. doi: 10.1111/jcmm.16021 (PMC7754003; doi:10.1111/jcmm.16021)
Supplement: Supplementary file 12 — Table S2 [file JCMM-24-14099-s012.docx]

**Supplementary Table 2.** Clinical features of CP/CPPS patients and healthy controls enrolled in BD™ AbSeq on the Rhapsody™ platform analysis.

| **Features** | **Control 1** | **Control 2** | **Case 1** | **Case 2** | ***P*-value^a^** |
| --- | --- | --- | --- | --- | --- |
| Lecithin corpuscle density | - | - | ++ | ++ |  |
| White cell in EPS | - | - | - | - |  |
| White cell in urine | - | - | - | - |  |
| NIH-CPSI | - | - | 23 | 17 |  |
| Age | 30 | 28 | 50 | 32 | 0.3162 |
| BMI | 19.22 | 28.59 | 22.49 | 17.99 | 0.5538 |

CP/CPPS, chronic prostatitis/chronic pelvic pain syndrome; EPS, expressed prostatic secretious; NIH-CPSI, The National Institutes of Health chronic prostatitis symptom index; BMI, Body Mass Index; ^a^, used unpaired t test.
