# Supplementary material for: Single‐cell multi‐omics analysis presents the landscape of peripheral blood T‐cell subsets in human chronic prostatitis/chronic pelvic pain syndrome
Source: J Cell Mol Med. 2020 Oct 30;24(23):14099–109. doi: 10.1111/jcmm.16021 (PMC7754003; doi:10.1111/jcmm.16021)
Supplement: Supplementary file 13 — Table S3 [file JCMM-24-14099-s013.docx]

**Supplementary table 3.** Cell viability of samples before performing single-cell sequencing.

| Group | Cell number | Cell viability (%) |
| --- | --- | --- |
| Healthy control | 1.63 x 10^6^ | 83% |
| CP/CPPS | 7.62x10^5^ | 76% |

CP/CPPS, chronic prostatitis/chronic pelvic pain symptom
