# Supplementary material for: Single‐cell multi‐omics analysis presents the landscape of peripheral blood T‐cell subsets in human chronic prostatitis/chronic pelvic pain syndrome
Source: J Cell Mol Med. 2020 Oct 30;24(23):14099–109. doi: 10.1111/jcmm.16021 (PMC7754003; doi:10.1111/jcmm.16021)
Supplement: Supplementary file 14 — Table S4 [file JCMM-24-14099-s014.doc]

**Supplementary table 4.** The annotation statistics of the sequence data.

| **Parameters** | **Control** | | |  | **Case** | | | |
| --- | --- | --- | --- | --- | --- | --- | --- | --- |
|  | **Multiplet** | **Sample tag05** | **Sample tag06** |  | **Multiplet** | **Undetermined** | **Sample tag7** | **Sample tag8** |
| Number of cells | 856 | 870 | 2510 |  | 5279 | 186 | 3216 | 6677 |
| Number of genes in annotated group | 274 | 274 | 274 |  | 274 | 274 | 274 | 274 |
| Number of genes detected | 258 | 249 | 259 |  | 262 | 200 | 255 | 261 |
| Mean molecules/cell | 40752 | 10450 | 17556 |  | 9834 | 1082 | 3834 | 4655 |
| Median molecules/cell | 37791 | 8828 | 12604 |  | 9119 | 720 | 2916 | 3548 |
| Max molecules/cell | 174096 | 126323 | 124118 |  | 62682 | 13070 | 45707 | 35169 |
| Min molecules/cell | 6204 | 701 | 2013 |  | 800 | 216 | 343 | 287 |
| Mean gene/cell | 111 | 76 | 84 |  | 110 | 33 | 82 | 86 |
| Median gene/cell | 111 | 76 | 80 |  | 111 | 32 | 79 | 83 |
| Max gene/cell | 165 | 124 | 154 |  | 164 | 77 | 150 | 161 |
| Min gene/cell | 41 | 39 | 34 |  | 33 | 22 | 26 | 28 |

Multiplet, means the cell containing two more tags that cannot be discriminated.
