# Supplementary material for: Single‐cell multi‐omics analysis presents the landscape of peripheral blood T‐cell subsets in human chronic prostatitis/chronic pelvic pain syndrome
Source: J Cell Mol Med. 2020 Oct 30;24(23):14099–109. doi: 10.1111/jcmm.16021 (PMC7754003; doi:10.1111/jcmm.16021)
Supplement: Supplementary file 15 — Table S5 [file JCMM-24-14099-s015.pdf]

Supplementary table 5. The differentially expressed genes between one cluster and others.

Gene	*P* _value	Average_logFoldChange	pct.1	pct.2	*P* _value_adjusted	Cluster
CD4	0.0000	0.4256	1.0000	1.0000	0.0000	0
CD69	0.0000	0.3559	0.9930	0.9140	0.0000	0
CD27	0.0000	0.3490	1.0000	1.0000	0.0000	0
CD52	0.0000	0.2941	1.0000	0.9780	0.0000	0
CD48	0.0000	0.2741	0.9990	0.9580	0.0000	0
CD3D	0.0000	0.2516	0.9990	0.9540	0.0000	0
CD27	0.0000	0.3132	0.8420	0.6200	0.0000	0
EGR1	0.0000	0.2937	0.8260	0.6570	0.0000	0
DUSP2	0.0000	0.3041	0.9480	0.8020	0.0000	0
CD25	0.0000	0.3054	0.9910	0.9600	0.0000	0
CD8A	0.0000	1.3010	0.9830	0.7790	0.0000	1
GZMB	0.0000	1.2911	0.9180	0.6800	0.0000	1
GZMH	0.0000	1.2766	0.9850	0.7420	0.0000	1
GNLY	0.0000	1.1763	0.9120	0.7500	0.0000	1
NKG7	0.0000	1.1552	1.0000	0.8660	0.0000	1
PRF1	0.0000	1.0214	0.8010	0.6240	0.0000	1
CCL5	0.0000	0.9510	1.0000	0.9050	0.0000	1
CST7	0.0000	0.9375	0.9730	0.8240	0.0000	1
LGALS1	0.0000	0.9135	0.8880	0.7830	0.0000	1
CD8	0.0000	0.8796	1.0000	1.0000	0.0000	1
KLRK1	0.0000	0.8747	0.9100	0.7780	0.0000	1
CTSW	0.0000	0.8580	0.9340	0.8260	0.0000	1
TARP	0.0000	0.8424	0.9300	0.8000	0.0000	1
GZMA	0.0000	0.8030	0.9010	0.7790	0.0000	1
RUNX3	0.0000	0.7808	0.9130	0.8620	0.0000	1
HLA-DPB1	0.0000	0.7744	0.8910	0.8050	0.0000	1
ITGAL	0.0000	0.7233	0.9350	0.9090	0.0000	1
ITGB2	0.0000	0.5970	0.9540	0.9480	0.0000	1
HLA-C	0.0000	0.4371	1.0000	0.9950	0.0000	1
HLA-A	0.0000	0.3230	1.0000	0.9980	0.0000	1
ZNF683	0.0000	1.1187	0.6370	0.4600	0.0000	1
CD8B	0.0000	0.8650	0.7500	0.6360	0.0000	1
HLA-DPA1	0.0000	0.7214	0.7700	0.6980	0.0000	1
TBX21	0.0000	0.7963	0.6700	0.5710	0.0000	1
IL32	0.0000	0.3000	0.9980	0.9840	0.0000	1
IL2RB	0.0000	0.5705	0.7490	0.7330	0.0000	1
HLA-DR	0.0000	0.3203	1.0000	0.9990	0.0000	1
TRAC	0.0000	0.3793	0.9860	0.9710	0.0000	1
SELPLG	0.0000	0.4955	0.7990	0.8430	0.0000	1
APOBEC3G	0.0000	0.6682	0.6770	0.6430	0.0000	1
CD52	0.0000	0.2795	0.9930	0.9820	0.0000	1
ITGAM	0.0000	0.7131	0.5350	0.4270	0.0000	1
GAPDH	0.0000	0.2689	0.9690	0.9680	0.0000	1
CX3CR1	0.0000	0.7072	0.4500	0.2980	0.0000	1
CD2	0.0000	0.4216	0.8400	0.8840	0.0000	1
BIN2	0.0000	0.3379	0.8680	0.9140	0.0000	1
KLRG1	0.0000	0.6149	0.6220	0.5830	0.0000	1
CCL4	0.0000	0.6101	0.5940	0.5400	0.0000	1
B3GAT1	0.0000	0.6628	0.4410	0.3300	0.0000	1
ARL4C	0.0000	0.4069	0.7370	0.7980	0.0000	1
IFNG	0.0000	0.7382	0.5460	0.4910	0.0000	1
CD300A	0.0000	0.5669	0.6070	0.5970	0.0000	1
FYN	0.0000	0.3535	0.7760	0.8480	0.0000	1
IER5	0.0000	0.3659	0.7900	0.8760	0.0000	1
HLA-DRB3	0.0000	0.5529	0.4910	0.4740	0.0000	1
TNFRSF1B	0.0000	0.4631	0.5660	0.5830	0.0000	1

EGR1	0.0000	0.8785	0.6740	0.7050	0.0000	1
LAIR2	0.0000	0.5931	0.4740	0.4690	0.0000	1
KLRC3	0.0000	0.4863	0.3500	0.3070	0.0000	1
ITGA4	0.0000	0.3733	0.6350	0.7100	0.0000	1
CD244	0.0000	0.5143	0.4170	0.3990	0.0000	1
HLA-DQA1	0.0000	0.3068	0.3180	0.2850	0.0000	1
CD45RA	0.0000	0.3018	1.0000	1.0000	0.0000	1
TSPAN32	0.0000	0.3883	0.5070	0.5610	0.0000	1
LAG3	0.0000	0.3874	0.4030	0.4110	0.0000	1
ANXA5	0.0000	0.3195	0.5810	0.6580	0.0000	1
TNF	0.0048	0.5687	0.4580	0.5080	1.0000	1
CLEC2D	0.0082	0.2646	0.4340	0.5430	1.0000	1
LEF1	0.0000	0.8946	0.9080	0.6390	0.0000	3
CD4	0.0000	0.8459	1.0000	1.0000	0.0000	3
CD27	0.0000	0.7190	1.0000	1.0000	0.0000	3
SELL	0.0000	0.7086	0.9270	0.7220	0.0000	3
PIK3IP1	0.0000	0.6140	0.9680	0.8350	0.0000	3
CD4	0.0000	0.7344	0.8500	0.6020	0.0000	3
CCR7	0.0000	0.7875	0.7900	0.5000	0.0000	3
CD38	0.0000	0.8126	0.9990	0.9890	0.0000	3
CD27	0.0000	0.6312	0.8610	0.6570	0.0000	3
FYB	0.0000	0.5486	0.9240	0.7990	0.0000	3
CD127	0.0000	0.2984	1.0000	0.9990	0.0000	3
TRIB2	0.0000	0.6113	0.7530	0.5710	0.0000	3
CD25	0.0000	0.6512	0.9890	0.9660	0.0000	3
IL7R	0.0000	0.3723	0.9380	0.8440	0.0000	3
LAT	0.0000	0.3514	0.9210	0.8380	0.0000	3
PASK	0.0000	0.5233	0.6040	0.4330	0.0000	3
TCF7	0.0000	0.4393	0.6200	0.4400	0.0000	3
CD69	0.0000	0.3658	0.9720	0.9300	0.0000	3
TRAT1	0.0000	0.4326	0.6650	0.5300	0.0000	3
TRBC2	0.0000	0.3186	0.9890	0.9750	0.0000	3
ICOS	0.0000	0.3827	0.5120	0.3770	0.0000	3
GIMAP5	0.0000	0.3393	0.7140	0.6160	0.0000	3
ITK	0.0000	0.2953	0.8230	0.7360	0.0000	3
GIMAP2	0.0000	0.2910	0.7950	0.7160	0.0000	3
TXK	0.0000	0.3292	0.4830	0.3530	0.0000	3
BCL11B	0.0000	0.3465	0.6750	0.5790	0.0000	3
STAT6	0.0000	0.2556	0.7920	0.7210	0.0000	3
LTB	0.0000	0.3267	0.4430	0.3170	0.0000	3
MYC	0.0000	0.3216	0.4700	0.3620	0.0000	3
IL4R	0.0000	0.2691	0.5580	0.4730	0.0000	3
FOXP3	0.0000	0.2676	0.3180	0.2290	0.0000	3
TNFRSF25	0.0000	0.3075	0.4680	0.3910	0.0000	3
CD4	0.0000	0.5239	1.0000	1.0000	0.0000	4
CD25	0.0000	0.5078	0.9980	0.9650	0.0000	4
CD127	0.0000	0.3624	1.0000	1.0000	0.0000	4
CD4	0.0000	0.4083	0.8210	0.6080	0.0000	4
IL7R	0.0000	0.3749	0.9610	0.8440	0.0000	4
CD196	0.0000	0.3268	0.9980	0.9930	0.0000	4
CCL20	0.0000	0.2621	0.2990	0.1350	0.0000	4
LTB	0.0000	0.2682	0.4830	0.3160	0.0000	4
GZMK	0.0000	1.0332	0.8190	0.5260	0.0000	5
CD8	0.0000	0.4893	1.0000	1.0000	0.0000	5
CCR5	0.0000	0.2844	0.4580	0.2520	0.0000	5
CCL5	0.0000	0.2508	0.9930	0.9150	0.0000	5
CD103	0.0000	1.0841	0.9790	0.9690	0.0000	5
DUSP2	0.0000	0.3140	0.8680	0.8380	0.0000	5
IL18RAP	0.0000	0.2546	0.5030	0.3620	0.0000	5

CXCR4	0.0000	0.2713	0.9410	0.9200	0.0000	5
CD196	0.0000	0.5403	0.9950	0.9930	0.0011	5
KLRB1	0.0000	0.4731	0.7300	0.6820	0.0021	5
TRDC	0.0000	1.8904	0.8540	0.3830	0.0000	6
TARP	0.0000	1.0015	0.9810	0.8120	0.0000	6
KLRB1	0.0000	1.3574	0.9430	0.6720	0.0000	6
CCL5	0.0000	0.6670	0.9990	0.9160	0.0000	6
CST7	0.0000	0.6930	0.9840	0.8410	0.0000	6
CD3	0.0000	0.5432	1.0000	1.0000	0.0000	6
CTSW	0.0000	0.6812	0.9540	0.8370	0.0000	6
HLA-A	0.0000	0.3577	1.0000	0.9980	0.0000	6
NKG7	0.0000	0.5865	0.9990	0.8820	0.0000	6
HLA-C	0.0000	0.3600	1.0000	0.9960	0.0000	6
IL18RAP	0.0000	0.9731	0.6130	0.3580	0.0000	6
GZMA	0.0000	0.6306	0.9230	0.7920	0.0000	6
DUSP2	0.0000	0.9914	0.9280	0.8350	0.0000	6
RUNX3	0.0000	0.4985	0.9290	0.8670	0.0000	6
KLRG1	0.0000	0.7898	0.7660	0.5800	0.0000	6
GZMK	0.0000	0.9338	0.7490	0.5330	0.0000	6
IL32	0.0000	0.3688	1.0000	0.9850	0.0000	6
IL2RB	0.0000	0.6190	0.8440	0.7310	0.0000	6
IER5	0.0000	0.6189	0.9120	0.8600	0.0000	6
DUSP1	0.0000	0.4561	0.9910	0.9830	0.0000	6
CD300A	0.0000	0.6881	0.7460	0.5920	0.0000	6
ITGB2	0.0000	0.3757	0.9670	0.9480	0.0000	6
IL7R	0.0000	0.6051	0.9300	0.8490	0.0000	6
JUN	0.0000	0.6378	0.8650	0.7940	0.0000	6
PRF1	0.0000	0.5341	0.7760	0.6460	0.0000	6
KLRK1	0.0000	0.4165	0.8910	0.7940	0.0000	6
ITGAL	0.0000	0.3310	0.9330	0.9120	0.0000	6
GAPDH	0.0000	0.2873	0.9920	0.9670	0.0000	6
TBX21	0.0000	0.5048	0.7150	0.5800	0.0000	6
KLRC1	0.0000	0.8124	0.4540	0.3010	0.0000	6
CCL4	0.0000	0.5657	0.6640	0.5430	0.0000	6
BIN2	0.0000	0.3321	0.9290	0.9060	0.0000	6
APOBEC3G	0.0000	0.4306	0.7300	0.6440	0.0000	6
GNLY	0.0000	0.6033	0.8090	0.7740	0.0000	6
ARL4C	0.0000	0.3968	0.8350	0.7860	0.0000	6
CD3D	0.0000	0.2532	0.9820	0.9650	0.0000	6
FYN	0.0000	0.3629	0.8620	0.8360	0.0000	6
LAG3	0.0000	0.5666	0.5330	0.4040	0.0000	6
HLA-DPB1	0.0000	0.2889	0.8560	0.8160	0.0000	6
IL12RB2	0.0000	0.3326	0.2640	0.1450	0.0000	6
LCK	0.0000	0.2784	0.9440	0.9260	0.0000	6
CD247	0.0000	0.4727	0.7130	0.6580	0.0000	6
NCR3	0.0000	0.6396	0.5040	0.3760	0.0000	6
EGR1	0.0000	1.0787	0.7600	0.6980	0.0000	6
ZBTB16	0.0000	0.6829	0.3140	0.1660	0.0000	6
CXCR4	0.0000	0.3402	0.9450	0.9200	0.0000	6
FOSB	0.0000	0.3359	0.9160	0.9240	0.0000	6
SELPLG	0.0000	0.2620	0.8310	0.8370	0.0000	6
CD44	0.0000	0.3218	0.7860	0.7730	0.0000	6
CD160	0.0000	0.3587	0.2790	0.1760	0.0000	6
CCR5	0.0000	0.3813	0.3370	0.2600	0.0000	6
CD7	0.0000	0.3764	0.6090	0.5810	0.0000	6
CBLB	0.0000	0.4134	0.5550	0.5290	0.0000	6
ITGAM	0.0000	0.2781	0.4880	0.4410	0.0000	6
KLRF1	0.0000	0.3436	0.3710	0.3170	0.0000	6
IL18R1	0.0000	0.4166	0.3690	0.3110	0.0000	6

CD244	0.0000	0.2674	0.4440	0.4000	0.0000	6
BAX	0.0000	0.2554	0.6850	0.7200	0.0000	6
SPOCK2	0.0000	0.3224	0.6300	0.6670	0.0002	6
ZAP70	0.0003	0.2579	0.5400	0.5610	0.0741	6
IFNGR1	0.0005	0.3061	0.3490	0.3330	0.1437	6
STAT4	0.0006	0.2703	0.4340	0.4420	0.1478	6
TNF	0.0006	0.6170	0.4820	0.5010	0.1523	6
CD45RA	0.0000	0.8393	1.0000	1.0000	0.0000	7
CCR7	0.0000	0.9537	0.8780	0.5120	0.0000	7
CD27	0.0000	0.6281	1.0000	1.0000	0.0000	7
LEF1	0.0000	0.9262	0.9480	0.6520	0.0000	7
CD8B	0.0000	0.8254	0.9380	0.6410	0.0000	7
PIK3IP1	0.0000	0.7097	0.9750	0.8420	0.0000	7
CD8	0.0000	0.5261	1.0000	1.0000	0.0000	7
SELL	0.0000	0.5376	0.9410	0.7330	0.0000	7
CD27	0.0000	0.5463	0.8920	0.6670	0.0000	7
TXK	0.0000	0.3702	0.6660	0.3520	0.0000	7
TCF7	0.0000	0.4472	0.7090	0.4460	0.0000	7
FYB	0.0000	0.3752	0.9230	0.8060	0.0000	7
CD7	0.0000	0.3699	0.7700	0.5740	0.0000	7
BCL11B	0.0000	0.3632	0.7790	0.5800	0.0000	7
KLRK1	0.0000	0.2515	0.9280	0.7930	0.0000	7
PASK	0.0000	0.3792	0.6590	0.4400	0.0000	7
TSPAN32	0.0000	0.2536	0.7450	0.5440	0.0000	7
CD38	0.0000	0.2663	0.9980	0.9900	0.0000	7
CD44	0.0000	0.4081	1.0000	1.0000	0.0000	8
GZMH	0.0000	0.6131	0.9880	0.7750	0.0000	9
GNLY	0.0000	0.6780	0.9590	0.7710	0.0000	9
CX3CR1	0.0000	0.3788	0.6210	0.3150	0.0000	9
NKG7	0.0000	0.3621	0.9980	0.8850	0.0000	9
CD4	0.0000	0.5500	0.8370	0.6210	0.0000	9
GZMA	0.0000	0.4158	0.9520	0.7940	0.0000	9
LGALS1	0.0000	0.3992	0.9420	0.7960	0.0000	9
B3GAT1	0.0000	0.3107	0.5640	0.3420	0.0000	9
CCL5	0.0000	0.2584	1.0000	0.9180	0.0000	9
RUNX3	0.0000	0.2765	0.9420	0.8680	0.0000	9
CD4	0.0000	0.3017	1.0000	1.0000	0.0000	9
TARP	0.0000	0.2638	0.9230	0.8180	0.0000	9
ARL4C	0.0000	0.3157	0.8490	0.7870	0.0000	9
ZNF683	0.0000	0.2984	0.6640	0.4840	0.0000	9
S1PR1	0.0000	0.2760	0.6450	0.4640	0.0000	9
SLAMF1	0.0000	0.2561	0.5490	0.3960	0.0000	9
APOBEC3G	0.0000	0.3062	0.7670	0.6460	0.0000	9
CD6	0.0000	0.2757	0.9210	0.8810	0.0000	9
TRDC	0.0000	2.4141	0.9760	0.3940	0.0000	10
NKG7	0.0000	1.5056	1.0000	0.8850	0.0000	10
CD45RA	0.0000	1.5270	1.0000	1.0000	0.0000	10
CST7	0.0000	1.4222	0.9890	0.8450	0.0000	10
TARP	0.0000	1.5042	0.9760	0.8170	0.0000	10
CD11c	0.0000	1.2838	1.0000	1.0000	0.0000	10
CCL5	0.0000	1.1089	1.0000	0.9180	0.0000	10
HLA-C	0.0000	0.7766	1.0000	0.9960	0.0000	10
CD3	0.0000	0.9927	1.0000	1.0000	0.0000	10
CTSW	0.0000	1.2725	0.9680	0.8400	0.0000	10
GZMH	0.0000	1.3121	0.9600	0.7760	0.0000	10
HLA-A	0.0000	0.6027	0.9970	0.9980	0.0000	10
ITGAL	0.0000	1.0209	0.9440	0.9120	0.0000	10
IL2RB	0.0000	1.4376	0.8730	0.7330	0.0000	10
GZMB	0.0000	1.4230	0.9050	0.7130	0.0000	10

KLRF1	0.0000	1.7233	0.6850	0.3130	0.0000	10
ITGB2	0.0000	0.9345	0.9600	0.9490	0.0000	10
PRF1	0.0000	1.5125	0.8490	0.6480	0.0000	10
IL32	0.0000	0.7496	1.0000	0.9860	0.0000	10
RUNX3	0.0000	1.0439	0.9150	0.8690	0.0000	10
GZMA	0.0000	1.0031	0.8920	0.7960	0.0000	10
HLA-DPB1	0.0000	0.8989	0.8860	0.8170	0.0000	10
CD123	0.0000	0.8043	0.9180	0.9560	0.0000	10
KLRK1	0.0000	0.9022	0.8680	0.7970	0.0000	10
CD3D	0.0000	0.6295	0.9760	0.9650	0.0000	10
ITGAX	0.0000	1.3667	0.4390	0.1640	0.0000	10
TBX21	0.0000	1.1610	0.7250	0.5840	0.0000	10
GAPDH	0.0000	0.5579	0.9740	0.9680	0.0000	10
BIN2	0.0000	0.7115	0.8680	0.9070	0.0000	10
HLA-DR	0.0000	0.4862	1.0000	0.9990	0.0000	10
KLRC3	0.0000	1.2182	0.5240	0.3100	0.0000	10
CD38	0.0000	0.5828	0.9840	0.9900	0.0000	10
HLA-DPA1	0.0000	0.8437	0.7280	0.7090	0.0000	10
GNLY	0.0000	1.1991	0.7830	0.7750	0.0000	10
FYN	0.0000	0.7866	0.7750	0.8380	0.0000	10
CCL4	0.0000	1.2710	0.6400	0.5470	0.0000	10
LCK	0.0000	0.5254	0.8760	0.9280	0.0000	10
KLRB1	0.0000	0.8714	0.7280	0.6840	0.0000	10
ARL4C	0.0000	0.7620	0.7300	0.7900	0.0000	10
LAG3	0.0000	1.0248	0.5240	0.4080	0.0000	10
CD300A	0.0000	0.9366	0.6350	0.5980	0.0000	10
CD247	0.0000	0.9344	0.6300	0.6610	0.0000	10
CCL3	0.0000	0.9256	0.3760	0.2490	0.0000	10
CD244	0.0000	0.9713	0.4950	0.4000	0.0000	10
IKZF2	0.0000	0.9078	0.3600	0.2440	0.0000	10
LAIR2	0.0000	1.1317	0.5340	0.4690	0.0000	10
CX3CR1	0.0000	0.9425	0.4390	0.3190	0.0000	10
CD3G	0.0000	0.4375	0.1930	0.4020	0.0000	10
SELPLG	0.0000	0.5403	0.7170	0.8390	0.0000	10
CD3E	0.0000	0.2824	0.9580	0.9730	0.0000	10
NCR3	0.0000	0.9238	0.4290	0.3800	0.0000	10
STAT4	0.0000	0.2585	0.2620	0.4450	0.0000	10
APOBEC3G	0.0000	0.7196	0.5660	0.6500	0.0001	10
LGALS1	0.0000	0.6273	0.6880	0.8010	0.0008	10
KLRC4	0.0001	0.5163	0.2860	0.2560	0.0297	10
KLRG1	0.0006	0.3162	0.3990	0.5930	0.1564	10
STAT3	0.0013	0.2773	0.3150	0.4560	0.3329	10
IFNG	0.0026	0.7729	0.4580	0.5010	0.6912	10
CBLB	0.0048	0.5218	0.3570	0.5340	1.0000	10
ITGAM	0.0052	0.5868	0.4210	0.4440	1.0000	10
TNFRSF1B	0.0055	0.5856	0.4970	0.5820	1.0000	10
TIAF1	0.0092	0.2840	0.3150	0.4510	1.0000	10
CD11c	0.0000	1.3391	1.0000	1.0000	0.0000	11
FOSB	0.0000	1.8860	0.9860	0.9220	0.0000	11
CD103	0.0000	1.3663	0.9750	0.9690	0.0000	11
CD196	0.0000	1.6141	0.9860	0.9930	0.0000	11
CD123	0.0000	1.4469	0.9140	0.9560	0.0000	11
CD45RO	0.0000	0.7210	1.0000	1.0000	0.0000	11
CBLB	0.0000	0.2660	0.1880	0.5370	0.0000	11
CD127	0.0000	0.4795	0.9970	1.0000	0.0000	11
IL18	0.0000	0.2512	0.1160	0.3150	0.0000	11
TXK	0.0000	0.2850	0.1660	0.3690	0.0000	11
RORA	0.0000	0.5171	0.2110	0.4120	0.0000	11
IL23R	0.0039	0.6744	0.2350	0.3890	1.0000	11

TYMS	0.0000	1.9015	0.6240	0.0960	0.0000	12
TK1	0.0000	1.5793	0.5990	0.1090	0.0000	12
TOP2A	0.0000	1.4264	0.4680	0.0670	0.0000	12
MKI67	0.0000	0.5130	0.2660	0.0260	0.0000	12
HLA-DR	0.0000	1.8819	1.0000	0.9990	0.0000	12
AURKB	0.0000	0.8742	0.4180	0.0610	0.0000	12
GAPDH	0.0000	1.3031	0.9920	0.9670	0.0000	12
HMGB2	0.0000	1.9121	0.8060	0.5100	0.0000	12
CTLA4	0.0000	1.1277	0.6410	0.2880	0.0000	12
IL32	0.0000	0.7521	1.0000	0.9860	0.0000	12
ANXA5	0.0000	1.1670	0.9030	0.6430	0.0000	12
UBE2C	0.0000	1.2133	0.3840	0.0720	0.0000	12
HLA-DRA	0.0000	0.9201	0.7930	0.4740	0.0000	12
PTTG2	0.0000	1.0498	0.5570	0.2200	0.0000	12
CD52	0.0000	0.5417	0.9960	0.9830	0.0000	12
DUSP4	0.0000	0.8279	0.4220	0.1520	0.0000	12
CD38	0.0000	1.0266	1.0000	0.9900	0.0000	12
TRAC	0.0000	0.4817	1.0000	0.9730	0.0000	12
SELPLG	0.0000	0.5171	0.9450	0.8350	0.0000	12
LGALS1	0.0000	0.9616	0.9320	0.7970	0.0000	12
CD4	0.0000	0.6126	0.8230	0.6230	0.0000	12
CD25	0.0000	1.2468	0.9790	0.9680	0.0000	12
ICOS	0.0000	0.6485	0.6030	0.3880	0.0000	12
CCNB1	0.0000	0.8613	0.3630	0.1430	0.0000	12
F5	0.0000	0.3029	0.3160	0.1300	0.0000	12
FOXP3	0.0000	0.8623	0.4260	0.2350	0.0000	12
CD45RO	0.0000	0.3609	1.0000	1.0000	0.0000	12
HLA-DMA	0.0000	0.4148	0.5700	0.3610	0.0000	12
TRIB2	0.0000	0.5563	0.7550	0.5870	0.0000	12
FAS	0.0000	0.4694	0.5530	0.3470	0.0000	12
CASP3	0.0000	0.4085	0.4890	0.2910	0.0000	12
HLA-DPA1	0.0000	0.4797	0.8020	0.7080	0.0000	12
ARL4C	0.0000	0.4578	0.8900	0.7870	0.0000	12
CD2	0.0000	0.3384	0.9450	0.8760	0.0000	12
CD44	0.0000	0.3505	0.8900	0.7720	0.0000	12
CD27	0.0000	0.6040	0.7640	0.6760	0.0000	12
TRBC2	0.0000	0.3725	1.0000	0.9760	0.0000	12
HLA-DRB3	0.0000	0.6635	0.5990	0.4760	0.0000	12
HLA-DQB1	0.0000	0.3171	0.4770	0.3180	0.0000	12
SELL	0.0000	0.6205	0.8480	0.7400	0.0000	12
ITGAE	0.0000	0.4853	0.6290	0.4840	0.0000	12
LCK	0.0000	0.3638	0.9830	0.9270	0.0000	12
TNFRSF1B	0.0000	0.4689	0.7130	0.5780	0.0000	12
CD4	0.0000	0.2925	1.0000	1.0000	0.0000	12
FYB	0.0000	0.3663	0.9030	0.8100	0.0000	12
S1PR1	0.0000	0.4409	0.6160	0.4660	0.0000	12
CD5	0.0000	0.3557	0.8060	0.6960	0.0000	12
LGALS3	0.0000	0.4757	0.4300	0.3020	0.0000	12
HLA-DPB1	0.0000	0.2883	0.8860	0.8170	0.0000	12
BAX	0.0000	0.3804	0.8230	0.7170	0.0000	12
TRAT1	0.0000	0.4165	0.6670	0.5420	0.0000	12
IL23R	0.0000	0.3603	0.5230	0.3850	0.0000	12
HLA-DQA1	0.0000	0.2935	0.4090	0.2890	0.0000	12
BIN2	0.0000	0.2545	0.9280	0.9060	0.0000	12
NINJ2	0.0000	0.2840	0.3920	0.2780	0.0000	12
TNFSF10	0.0000	0.3083	0.6120	0.4740	0.0000	12
LAP3	0.0000	0.4045	0.5400	0.3970	0.0000	12
PRDM1	0.0000	0.2587	0.3840	0.2750	0.0001	12
CLEC2D	0.0000	0.3136	0.6410	0.5250	0.0001	12

CXCR3	0.0000	0.7652	0.4430	0.3190	0.0001	12
ITK	0.0000	0.2556	0.8230	0.7430	0.0007	12
STAT1	0.0000	0.3129	0.5360	0.4400	0.0016	12
TIGIT	0.0000	0.3246	0.3460	0.2410	0.0035	12
CCND2	0.0000	0.3508	0.5060	0.4220	0.0117	12
IRF4	0.0001	0.2947	0.3040	0.2200	0.0166	12
LEF1	0.0001	0.2838	0.7300	0.6630	0.0314	12
IL2RA	0.0004	0.3130	0.3120	0.2440	0.0967	12
LILRB4	0.0000	0.4763	0.4630	0.0020	0.0000	13
CXCL8	0.0000	2.9205	0.9000	0.2100	0.0000	13
HLA-DMB	0.0000	1.7249	0.9260	0.2300	0.0000	13
TLR2	0.0000	0.7191	0.5800	0.0480	0.0000	13
HLA-DRA	0.0000	3.2389	0.9960	0.4710	0.0000	13
LAT2	0.0000	1.2262	0.7840	0.1430	0.0000	13
ITGAX	0.0000	1.1488	0.7880	0.1620	0.0000	13
HLA-DMA	0.0000	1.7267	0.9480	0.3570	0.0000	13
ENTPD1	0.0000	0.4593	0.4940	0.0250	0.0000	13
IRF8	0.0000	1.0378	0.7100	0.1350	0.0000	13
CCL3	0.0000	2.1029	0.8270	0.2450	0.0000	13
HLA-DQB1	0.0000	1.8611	0.8740	0.3140	0.0000	13
HLA-DR	0.0000	2.2577	1.0000	0.9990	0.0000	13
CD11c	0.0000	2.2626	1.0000	1.0000	0.0000	13
LGALS3	0.0000	1.2971	0.8440	0.2970	0.0000	13
NAMPT	0.0000	1.1002	0.7580	0.2290	0.0000	13
HLA-DPA1	0.0000	1.7177	0.9960	0.7060	0.0000	13
HLA-DRB3	0.0000	1.9293	0.8920	0.4720	0.0000	13
IER3	0.0000	1.3468	0.6710	0.2180	0.0000	13
HLA-DQA1	0.0000	1.4639	0.7320	0.2850	0.0000	13
ANXA5	0.0000	1.1178	0.9570	0.6430	0.0000	13
HLA-DPB1	0.0000	1.2729	1.0000	0.8160	0.0000	13
C10orf54	0.0000	1.1533	0.9390	0.6400	0.0000	13
CD38	0.0000	0.7336	1.0000	0.9900	0.0000	13
GAPDH	0.0000	0.8416	0.9960	0.9670	0.0000	13
PECAM1	0.0000	0.5759	0.5280	0.1250	0.0000	13
LGALS1	0.0000	0.7973	0.9520	0.7970	0.0000	13
VNN2	0.0000	0.6045	0.6970	0.3260	0.0000	13
ITGB2	0.0000	0.8957	0.9910	0.9490	0.0000	13
HAVCR2	0.0000	0.2901	0.4550	0.1390	0.0000	13
ICAM1	0.0000	0.5865	0.5840	0.2510	0.0000	13
CD123	0.0000	0.7312	1.0000	0.9540	0.0000	13
JUN	0.0000	0.7760	0.9830	0.7950	0.0000	13
FOSB	0.0000	0.6485	0.9960	0.9220	0.0000	13
IFNGR1	0.0000	0.4986	0.6620	0.3300	0.0000	13
TNFRSF1B	0.0000	0.6854	0.8570	0.5770	0.0000	13
EGR1	0.0000	0.5566	0.9520	0.6980	0.0000	13
CD4	0.0000	0.6452	0.8920	0.6220	0.0000	13
TNFSF10	0.0000	0.6469	0.7750	0.4720	0.0000	13
ITGAM	0.0000	0.6152	0.7620	0.4400	0.0000	13
DUSP1	0.0000	0.6586	0.9960	0.9840	0.0000	13
BCL6	0.0000	0.2933	0.4550	0.2010	0.0000	13
LAP3	0.0000	0.5873	0.6710	0.3950	0.0000	13
IL18	0.0000	0.4090	0.5630	0.3090	0.0000	13
SELL	0.0000	0.5288	0.9610	0.7390	0.0000	13
STAT6	0.0000	0.4215	0.9220	0.7250	0.0000	13
BAX	0.0000	0.2524	0.9350	0.7160	0.0000	13
CD44	0.0000	0.4148	0.9440	0.7710	0.0000	13
CCR2	0.0000	0.4789	0.3720	0.1430	0.0000	13
CXCR1	0.0000	2.1533	0.7630	0.0610	0.0000	14
CXCL8	0.0000	5.0020	0.9800	0.2100	0.0000	14

NAMPT	0.0000	2.9316	0.9440	0.2280	0.0000	14
ITGAX	0.0000	2.2294	0.8330	0.1630	0.0000	14
CD11c	0.0000	2.5482	1.0000	1.0000	0.0000	14
TLR2	0.0000	1.1538	0.4850	0.0490	0.0000	14
VNN2	0.0000	2.1312	0.8180	0.3260	0.0000	14
DUSP1	0.0000	1.4107	1.0000	0.9840	0.0000	14
LAT2	0.0000	1.3651	0.5710	0.1460	0.0000	14
SELL	0.0000	1.2789	0.9600	0.7390	0.0000	14
C10orf54	0.0000	1.3889	0.8740	0.6420	0.0000	14
HLA-C	0.0000	0.6378	1.0000	0.9960	0.0000	14
FOSB	0.0000	0.9242	0.9750	0.9230	0.0000	14
CD45RO	0.0000	0.6589	1.0000	1.0000	0.0000	14
ICAM1	0.0000	1.2775	0.4800	0.2530	0.0000	14
SELPLG	0.0000	0.7537	0.8690	0.8360	0.0000	14
TNFRSF1B	0.0000	0.8106	0.7020	0.5790	0.0000	14
IFNGR1	0.0000	0.8514	0.5100	0.3320	0.0000	14
CD123	0.0000	0.6411	0.9490	0.9550	0.0000	14
CD38	0.0000	0.2695	0.9950	0.9900	0.0000	14
CD300A	0.0000	0.6811	0.6970	0.5980	0.0000	14
FYB	0.0000	0.6236	0.8280	0.8110	0.0000	14
STAT6	0.0000	0.6645	0.7580	0.7270	0.0000	14
HLA-A	0.0000	0.2907	1.0000	0.9980	0.0000	14
ITGB2	0.0000	0.3231	0.9240	0.9500	0.0000	14
PECAM1	0.0000	0.6378	0.2530	0.1290	0.0001	14
TNFSF10	0.0001	0.8485	0.5100	0.4750	0.0207	14
BIN2	0.0002	0.3298	0.8640	0.9070	0.0492	14
JUNB	0.0004	0.4466	0.8280	0.8830	0.1119	14
STAT3	0.0019	0.5626	0.4750	0.4530	0.5085	14
IER3	0.0019	0.8425	0.2680	0.2230	0.5095	14

Note: there was no significant differentially expressed genes between cluster 2 and others.
