# Supplementary material for: Single‐cell multi‐omics analysis presents the landscape of peripheral blood T‐cell subsets in human chronic prostatitis/chronic pelvic pain syndrome
Source: J Cell Mol Med. 2020 Oct 30;24(23):14099–109. doi: 10.1111/jcmm.16021 (PMC7754003; doi:10.1111/jcmm.16021)
Supplement: Supplementary file 16 — Table S6 [file JCMM-24-14099-s016.pdf]

Supplementary table 6. Differentially expressed genes between cells derived from prostatitis patients and healthy contr

Cluster ID	GeneName	*P* _value	Average_logFoldChange	*P* _value_adjusted
Cluster 0	CTSW-NM-001335.3-Reference-end	4.09000E-27	0.32657	1.09000E-24
	PIK3IP1-NM-052880.4-Reference-end	3.46000E-25	0.31392	9.21000E-23
	ICOS-NM-012092.3-Reference-end	1.00000E-24	0.25414	2.67000E-22
	FYB-NM-001465.4-Reference-end	3.86000E-20	0.29653	1.03000E-17
	SELL-NM-000655.4-Reference-end	6.41000E-17	0.30198	1.71000E-14
	GZMB-NM-004131.4-Reference-end	2.29000E-16	0.42240	6.09000E-14
	CD8A-NM-001768.6-Reference-end	9.74000E-16	0.30891	2.59000E-13
	CD27-NM-001242.4-Reference-end	3.00000E-15	0.26140	7.98000E-13
	LEF1-NM-016269.4-Reference-end	1.27000E-14	0.35293	3.38000E-12
	GIMAP2-NM-015660.2-Reference-end	1.58000E-14	0.26655	4.20000E-12
	ITK-NM-005546.3-Reference-end	3.95000E-14	0.26700	1.05000E-11
	LGALS1-NM-002305.3-Reference-end	5.03000E-14	0.34269	1.34000E-11
	CD103-ITGAE-AHS0001-pAbO	2.00000E-12	0.56094	5.31000E-10
	STAT6-NM-003153.4-Reference-end	6.58000E-10	0.25007	1.75000E-07
	HLA-DRA-NM-019111.4-Reference-end	5.47000E-09	0.29238	1.45000E-06
	ANXA5-NM-001154.3-Reference-end	1.15000E-07	0.27563	3.05000E-05
	SPOCK2-NM-014767.2-Reference-end	2.87000E-07	0.29335	7.63000E-05
	C10orf54-NM-022153.1-PolyA-1	3.83000E-07	0.25983	1.01837E-04
	CD8B-NM-004931.4-Reference-end	1.03000E-05	0.34783	2.73246E-03
	GNLY-NM-006433.4-Reference-end	1.16922E-04	0.27929	3.11013E-02
Cluster 1	TRDC-ENST00000390477.2-Reference-er	0.00000E+00	0.25609	0.00000E+00
	HLA-DQA1-NM-002122.3-PolyA-1	1.13000E-202	0.33179	3.01000E-200
	GZMK-NM-002104.2-Reference-end	1.05000E-196	0.37039	2.80000E-194
	STAT1-NM-007315.3-Reference-end	2.40000E-173	0.33983	6.37000E-171
	RORA-NM-002943.3-Reference-end	3.48000E-169	0.25043	9.26000E-167
	IL18R1-NM-003855.3-Reference-end	3.64000E-166	0.26219	9.69000E-164
	NCR3-NM-147130.2-Reference-end	7.49000E-146	0.28171	1.99000E-143
	KLRB1-NM-002258.2-Reference-end	4.33000E-118	0.47105	1.15000E-115
	TRIB2-NM-021643.3-Reference-end	1.19000E-112	0.32718	3.18000E-110
	STAT3-NM-003150.3-Reference-end	2.47000E-111	0.31853	6.58000E-109
	BCL11B-NM-022898.2-Reference-end	5.93000E-81	0.34089	1.58000E-78
	STAT4-NM-003151.3-Reference-end	1.08000E-77	0.26646	2.87000E-75
	CBLB-NM-170662.4-PolyA-1	4.07000E-74	0.27384	1.08000E-71
	IL18RAP-NM-003853.3-Reference-end	3.73000E-69	0.31188	9.92000E-67
	TNF-NM-000594.3-Reference-end	3.43000E-64	0.35816	9.13000E-62
	IL4R-NM-000418.3-Reference-end	2.08000E-60	0.27140	5.53000E-58
	LAG3-NM-002286.5-Reference-end	2.32000E-53	0.31768	6.17000E-51
	HLA-DRA-NM-019111.4-Reference-end	5.66000E-51	0.48633	1.51000E-48
	CD103-ITGAE-AHS0001-pAbO	5.03000E-47	0.69271	1.34000E-44
	B3GAT1-NM-018644.3-Reference-end	1.57000E-40	0.40508	4.18000E-38
	SPOCK2-NM-014767.2-Reference-end	1.20000E-36	0.30683	3.19000E-34
	C10orf54-NM-022153.1-PolyA-1	4.72000E-35	0.29642	1.25000E-32
	HLA-DRB3-NM-022555.3-Reference-end	8.52000E-33	0.46737	2.27000E-30
	STAT5A-NM-003152.3-Reference-end	6.39000E-29	0.29779	1.70000E-26
	KLRC4-NM-013431.2-Reference-end	4.72000E-27	0.28199	1.26000E-24
	TARP-refseq-NM-001003799.1-Reference	1.23000E-24	0.38742	3.27000E-22
	CD5-NM-014207.3-Reference-end	4.51000E-20	0.32060	1.20000E-17
	CTSW-NM-001335.3-Reference-end	1.78000E-19	0.30051	4.73000E-17
	IFNG-NM-000619.2-Reference-end	4.27000E-18	0.32811	1.13000E-15
	CD196-CCR6-AHS0034-pAbO	5.31000E-17	0.50704	1.41000E-14
	RUNX3-NM-004350.2-Reference-end	1.14000E-15	0.30329	3.02000E-13
	IL12RB1-NM-005535.2-Reference-end	2.10000E-15	0.28564	5.58000E-13
	HLA-DPB1-NM-002121.5-PolyA-1	2.57000E-14	0.32381	6.85000E-12
	CD244-NM-016382.3-Reference-end	6.36000E-14	0.39372	1.69000E-11
	CD123-IL3RA-AHS0020-pAbO	1.74000E-13	0.32802	4.62000E-11
	CX3CR1-NM-001337.3-Reference-end	3.70000E-11	0.31832	9.84000E-09
	KLRK1-NM-007360.3-Reference-end	6.61000E-11	0.26582	1.76000E-08
	TNFRSF1B-NM-001066.2-Reference-end	1.27000E-09	0.34744	3.37000E-07
	FYN-NM-002037.5-PolyA-1	2.50000E-09	0.41154	6.65000E-07
	BAX-NM-001291428.1-Reference-end	3.13000E-09	0.28257	8.31000E-07
	IL7R-NM-002185.3-Reference-end	1.04000E-08	0.48001	2.78000E-06
	CD69-NM-001781.2-Reference-end	2.34000E-08	0.27700	6.24000E-06
	HLA-DPA1-NM-033554.3-PolyA-1	1.24000E-07	0.40005	3.29000E-05
	LGALS1-NM-002305.3-Reference-end	1.88000E-07	0.27274	5.01000E-05
	ANXA5-NM-001154.3-Reference-end	4.75000E-07	0.41196	1.26246E-04
	CD247-NM-000734.3-Reference-end	1.62000E-06	0.38174	4.30062E-04
	FYB-NM-001465.4-Reference-end	4.17000E-05	0.33006	1.10938E-02

	CD300A-NM-007261.3-Reference-end	4.54000E-05	0.32217	1.20813E-02
	PIK3IP1-NM-052880.4-Reference-end	1.54009E-04	0.26871	4.09664E-02
Cluster 2	CD8A-NM-001768.6-Reference-end	1.15000E-36	0.41149	3.06000E-34
	FYB-NM-001465.4-Reference-end	1.30000E-28	0.31944	3.45000E-26
	ITGAL-NM-002209.2-Reference-end	5.38000E-28	0.28778	1.43000E-25
	PIK3IP1-NM-052880.4-Reference-end	4.92000E-21	0.25182	1.31000E-18
	GNLY-NM-006433.4-Reference-end	2.09000E-16	0.38820	5.55000E-14
	GZMB-NM-004131.4-Reference-end	4.19000E-15	0.33547	1.11000E-12
	SELL-NM-000655.4-Reference-end	1.35000E-13	0.27416	3.60000E-11
	ZNF683-NM-173574.3-Reference-end	9.82000E-10	0.25504	2.61000E-07
	CD8B-NM-004931.4-Reference-end	3.10000E-09	0.32751	8.23000E-07
	CD38-CD38-AHS0022-pAbO	3.55000E-09	-0.36885	9.44000E-07
	LEF1-NM-016269.4-Reference-end	5.04000E-08	0.27479	1.34000E-05
	CD25-IL2RA-AHS0026-pAbO	6.39000E-08	-0.26636	1.70000E-05
	CD103-ITGAE-AHS0001-pAbO	6.44000E-06	-0.37681	1.71387E-03
	ITGA4-NM-000885.5-PolyA-1	9.69000E-06	0.26169	2.57631E-03
	APOBEC3G-NM-021822.3-Reference-end	3.66000E-05	0.26155	9.73417E-03
	SPOCK2-NM-014767.2-Reference-end	1.14196E-04	0.26799	3.03763E-02
Cluster 3	GZMK-NM-002104.2-Reference-end	1.22000E-106	0.33689	3.24000E-104
	GNLY-NM-006433.4-Reference-end	8.10000E-86	0.33112	2.15000E-83
	RORA-NM-002943.3-Reference-end	1.40000E-63	0.25772	3.72000E-61
	CCND2-NM-001759.3-Reference-end	1.08000E-50	0.27101	2.86000E-48
	CBLB-NM-170662.4-PolyA-1	7.07000E-41	0.25572	1.88000E-38
	STAT1-NM-007315.3-Reference-end	1.20000E-35	0.26645	3.18000E-33
	STAT3-NM-003150.3-Reference-end	1.58000E-32	0.31742	4.19000E-30
	TXK-NM-003328.2-Reference-end	7.72000E-32	0.35660	2.05000E-29
	CLEC2D-NM-013269.5-PolyA-1	1.52000E-31	0.26283	4.04000E-29
	EGR1-NM-001964.2-Reference-end	2.28000E-31	0.63630	6.07000E-29
	NKG7-NM-005601.3-Reference-end	1.65000E-28	0.45369	4.40000E-26
	CD11c-ITGAX-AHS0056-pAbO	1.27000E-27	0.46975	3.38000E-25
	S1PR1-NM-001400.4-Reference-end	3.63000E-21	0.25869	9.65000E-19
	FYB-NM-001465.4-Reference-end	6.91000E-19	0.36140	1.84000E-16
	TNFRSF1B-NM-001066.2-Reference-end	1.46000E-18	0.32776	3.88000E-16
	IL2RB-NM-000878.3-Reference-end	8.12000E-17	0.30483	2.16000E-14
	HLA-DPB1-NM-002121.5-PolyA-1	8.14000E-16	0.29423	2.17000E-13
	CD196-CCR6-AHS0034-pAbO	8.53000E-11	0.25195	2.27000E-08
	LEF1-NM-016269.4-Reference-end	9.85000E-10	0.34709	2.62000E-07
	SELPLG-NM-003006.4-PolyA-1	3.34000E-08	0.40639	8.89000E-06
	CD123-IL3RA-AHS0020-pAbO	3.59000E-08	0.31029	9.54000E-06
	ITGAL-NM-002209.2-Reference-end	1.34000E-07	0.25825	3.56000E-05
	IL23R-NM-144701.2-Reference-end	5.06000E-07	0.28937	1.34546E-04
	IER5-NM-016545.4-Reference-end	6.30000E-07	0.39251	1.67567E-04
	FOSB-NM-006732.2-Reference-end	6.72000E-07	0.32368	1.78625E-04
	CCL5-NM-002985.2-Reference-end	1.12000E-06	0.27110	2.98792E-04
	KLRB1-NM-002258.2-Reference-end	2.96000E-06	0.30928	7.87213E-04
	CD4-NM-000616.4-Reference-end	7.82000E-06	0.26187	2.07906E-03
	FYN-NM-002037.5-PolyA-1	9.36000E-06	0.29158	2.48923E-03
	ITK-NM-005546.3-Reference-end	1.69000E-05	0.26020	4.49619E-03
	CCR7-NM-001838.3-Reference-end	3.84000E-05	0.35797	1.02202E-02
Cluster 4	CD103-ITGAE-AHS0001-pAbO	1.28000E-16	-0.65947	3.41000E-14
	CD300A-NM-007261.3-Reference-end	2.76000E-55	0.26983	7.34000E-53
	TNF-NM-000594.3-Reference-end	4.67000E-50	0.33188	1.24000E-47
	CD8A-NM-001768.6-Reference-end	3.87000E-44	0.26023	1.03000E-41
	RORA-NM-002943.3-Reference-end	5.88000E-44	0.26816	1.57000E-41
	CBLB-NM-170662.4-PolyA-1	2.00000E-42	0.28898	5.32000E-40
	GNLY-NM-006433.4-Reference-end	2.05000E-42	0.40113	5.46000E-40
	APOBEC3G-NM-021822.3-Reference-end	2.78000E-38	0.27937	7.39000E-36
	EGR1-NM-001964.2-Reference-end	8.96000E-35	0.75448	2.38000E-32
	MYC-NM-002467.4-Reference-end	3.15000E-32	0.25133	8.37000E-30
	CD11c-ITGAX-AHS0056-pAbO	3.46000E-24	0.44017	9.21000E-22
	SLAMF1-ENST00000302035.10-PolyA-1	4.32000E-23	0.26646	1.15000E-20
	ZAP70-NM-001079.3-Reference-end	4.09000E-20	0.26823	1.09000E-17
	TARP-refseq-NM-001003799.1-Reference	6.69000E-19	0.34714	1.78000E-16
	BTG1-NM-001731.2-PolyA-1	1.38000E-17	0.25508	3.68000E-15
	CCR7-NM-001838.3-Reference-end	3.47000E-16	0.30389	9.23000E-14
	TNFSF10-NM-003810.3-Reference-end	7.27000E-15	0.26038	1.93000E-12
	GZMK-NM-002104.2-Reference-end	7.88000E-15	0.47235	2.10000E-12
	GZMA-NM-006144.3-Reference-end	3.12000E-12	0.39536	8.30000E-10
	TSPAN32-NM-139022.2-Reference-end	9.89000E-11	0.26559	2.63000E-08
	FOSB-NM-006732.2-Reference-end	3.63000E-10	0.39477	9.66000E-08

	FYB-NM-001465.4-Reference-end	6.17000E-10	0.36674	1.64000E-07
	ITGAL-NM-002209.2-Reference-end	3.25000E-09	0.33342	8.63000E-07
	CST7-NM-003650.3-Reference-end	4.86000E-08	0.35287	1.29000E-05
	CD123-IL3RA-AHS0020-pAbO	3.81000E-07	0.32595	1.01387E-04
	CCL5-NM-002985.2-Reference-end	1.88000E-06	0.57625	4.99825E-04
	CTSW-NM-001335.3-Reference-end	6.56000E-06	0.36326	1.74459E-03
	IL2RB-NM-000878.3-Reference-end	2.51000E-05	0.33363	6.67003E-03
Cluster 5	ZNF683-NM-173574.3-Reference-end	9.78000E-96	0.29980	2.60000E-93
	HLA-DQB1-NM-002123.4-PolyA-1	1.57000E-89	0.27693	4.18000E-87
	KLRC1-NM-002259.4-Reference-end	6.26000E-76	0.34712	1.67000E-73
	IL4R-NM-000418.3-Reference-end	4.48000E-63	0.25392	1.19000E-60
	GZMB-NM-004131.4-Reference-end	1.12000E-41	0.32059	2.99000E-39
	TRDC-ENST00000390477.2-Reference-er	1.15000E-39	0.83403	3.05000E-37
	IL18RAP-NM-003853.3-Reference-end	9.78000E-35	0.35815	2.60000E-32
	TSPAN32-NM-139022.2-Reference-end	1.85000E-20	0.32037	4.92000E-18
	CD247-NM-000734.3-Reference-end	4.94000E-18	0.31497	1.31000E-15
	KLRG1-NM-005810.3-Reference-end	7.15000E-18	0.25968	1.90000E-15
	CD300A-NM-007261.3-Reference-end	1.64000E-15	0.50731	4.35000E-13
	GIMAP5-NM-018384.4-Reference-end	4.41000E-14	0.37884	1.17000E-11
	CD103-ITGAE-AHS0001-pAbO	7.73000E-14	-0.29826	2.06000E-11
	LGALS1-NM-002305.3-Reference-end	1.88000E-13	0.27283	5.01000E-11
	CD7-NM-006137.6-Reference-end	5.27000E-13	0.25646	1.40000E-10
	CD5-NM-014207.3-Reference-end	1.21000E-11	0.37070	3.21000E-09
	CTSW-NM-001335.3-Reference-end	9.13000E-11	0.37115	2.43000E-08
	BAX-NM-001291428.1-Reference-end	1.86000E-10	0.26217	4.96000E-08
	SPOCK2-NM-014767.2-Reference-end	1.06000E-09	0.28998	2.81000E-07
	CBLB-NM-170662.4-PolyA-1	9.47000E-09	0.49136	2.52000E-06
	SELL-NM-000655.4-Reference-end	9.61000E-09	0.39116	2.56000E-06
	EGR1-NM-001964.2-Reference-end	4.42000E-07	0.88081	1.17642E-04
	TARP-refseq-NM-001003799.1-Reference	8.69000E-07	0.48551	2.31070E-04
	HLA-DPA1-NM-033554.3-PolyA-1	8.76000E-07	0.25448	2.32987E-04
	TBX21-NM-013351.1-Reference-end	2.13000E-06	0.37364	5.65341E-04
	BIN2-NM-016293.3-Reference-end	8.34000E-06	0.39918	2.21916E-03
	CD69-NM-001781.2-Reference-end	7.48000E-05	0.26859	1.99021E-02
Cluster 6	SELL-NM-000655.4-Reference-end	9.27000E-05	-0.29424	2.46539E-02
	CD196-CCR6-AHS0034-pAbO	5.91000E-09	-0.52874	1.57000E-06
	CD3-CD3E-AHS0033-pAbO	3.68000E-09	-0.25051	9.78000E-07
	CD27-CD27-AHS0025-pAbO	3.78000E-12	-0.55993	1.01000E-09
	DPP4-NM-001935.3-Reference-end	5.74000E-49	0.27454	1.53000E-46
	HLA-DQB1-NM-002123.4-PolyA-1	1.05000E-36	0.34241	2.78000E-34
	BCL2-NM-000633.2-Reference-end	6.35000E-30	0.30474	1.69000E-27
	KLRC4-NM-013431.2-Reference-end	1.14000E-27	0.25942	3.03000E-25
	KLRC3-NM-002261.2-Reference-end	8.08000E-24	0.33902	2.15000E-21
	STAT5A-NM-003152.3-Reference-end	7.29000E-23	0.25721	1.94000E-20
	KLRF1-NM-016523.2-Reference-end	1.68000E-20	0.38350	4.47000E-18
	HLA-DRB3-NM-022555.3-Reference-end	6.73000E-19	0.34856	1.79000E-16
	HLA-DRA-NM-019111.4-Reference-end	1.38000E-17	0.26555	3.67000E-15
	TRAT1-NM-016388.3-Reference-end	1.26000E-13	0.25916	3.34000E-11
	HMGB2-NM-002129.3-PolyA-1	1.23000E-12	0.26769	3.26000E-10
	ITGAE-NM-002208.4-Reference-end	3.05000E-12	0.31214	8.10000E-10
	CD244-NM-016382.3-Reference-end	8.42000E-12	0.26209	2.24000E-09
	MYC-NM-002467.4-Reference-end	2.44000E-07	0.40694	6.50000E-05
	IL18R1-NM-003855.3-Reference-end	3.01000E-06	0.30657	7.99691E-04
	BCL11B-NM-022898.2-Reference-end	4.95000E-06	0.32171	1.31731E-03
	CD8B-NM-004931.4-Reference-end	5.66000E-06	0.45525	1.50655E-03
	TNF-NM-000594.3-Reference-end	1.62409E-04	0.31686	4.32008E-02
Cluster 7	GZMK-NM-002104.2-Reference-end	9.43000E-65	0.48510	2.51000E-62
	FOXO3-NM-001455.3-Reference-end	7.83000E-53	0.26532	2.08000E-50
	GNLY-NM-006433.4-Reference-end	1.44000E-52	0.26194	3.83000E-50
	CST7-NM-003650.3-Reference-end	7.78000E-48	0.32446	2.07000E-45
	LGALS1-NM-002305.3-Reference-end	8.48000E-39	0.32354	2.26000E-36
	KLRB1-NM-002258.2-Reference-end	8.71000E-39	0.68578	2.32000E-36
	ITGAE-NM-002208.4-Reference-end	1.59000E-38	0.25332	4.23000E-36
	TARP-refseq-NM-001003799.1-Reference	2.51000E-34	0.26044	6.66000E-32
	STAT3-NM-003150.3-Reference-end	4.83000E-29	0.25310	1.28000E-26
	CLEC2D-NM-013269.5-PolyA-1	3.36000E-27	0.30972	8.93000E-25
	TNFSF10-NM-003810.3-Reference-end	6.23000E-26	0.29157	1.66000E-23
	CNOT2-NM-014515.5-Reference-end	1.54000E-24	0.31232	4.11000E-22
	FOSB-NM-006732.2-Reference-end	3.20000E-23	0.69792	8.52000E-21
	CD45RO-PTPRC-AHS0036-pAbO	6.48000E-21	0.40807	1.72000E-18

	DUSP1-NM-004417.3-Reference-end	2.70000E-18	0.40433	7.18000E-16
	CD4-NM-000616.4-Reference-end	3.31000E-15	0.27725	8.81000E-13
	IL4R-NM-000418.3-Reference-end	9.51000E-15	0.28223	2.53000E-12
	NKG7-NM-005601.3-Reference-end	1.67000E-14	0.54344	4.45000E-12
	EGR1-NM-001964.2-Reference-end	4.22000E-14	0.88312	1.12000E-11
	TXK-NM-003328.2-Reference-end	7.86000E-13	0.36171	2.09000E-10
	CD6-NM-006725.4-Reference-end	2.37000E-10	0.31170	6.31000E-08
	STAT5A-NM-003152.3-Reference-end	4.52000E-10	0.32588	1.20000E-07
	FYB-NM-001465.4-Reference-end	5.62000E-10	0.34636	1.50000E-07
	IL2RB-NM-000878.3-Reference-end	6.36000E-10	0.36394	1.69000E-07
	CD69-NM-001781.2-Reference-end	1.13000E-08	0.32396	3.00000E-06
	CBLB-NM-170662.4-PolyA-1	1.46000E-08	0.44077	3.88000E-06
	IER5-NM-016545.4-Reference-end	3.20000E-08	0.49306	8.51000E-06
	ARL4C-NM-005737.3-Reference-end	3.24000E-08	0.27286	8.62000E-06
	HLA-DPB1-NM-002121.5-PolyA-1	4.09000E-08	0.41577	1.09000E-05
	IL7R-NM-002185.3-Reference-end	1.35000E-07	0.29166	3.59000E-05
	MYC-NM-002467.4-Reference-end	4.50000E-07	0.32751	1.19644E-04
	CD103-ITGAE-AHS0001-pAbO	1.52000E-06	0.45803	4.05022E-04
	DUSP2-NM-004418.3-Reference-end	1.96000E-06	0.54671	5.20322E-04
	TRIB2-NM-021643.3-Reference-end	1.97000E-06	0.42872	5.24866E-04
	ITGAL-NM-002209.2-Reference-end	2.77000E-05	0.32466	7.36942E-03
	CD196-CCR6-AHS0034-pAbO	3.14000E-05	0.73450	8.35501E-03
	JUNB-NM-002229.2-Reference-end	1.32411E-04	0.28902	3.52212E-02
Cluster 8	CD27-CD27-AHS0025-pAbO	3.95000E-06	-0.26878	1.05196E-03
	CD4-CD4-AHS0032-pAbO	4.13000E-06	-0.37441	1.09952E-03
	NKG7-NM-005601.3-Reference-end	1.78000E-05	0.34090	4.72754E-03
	CCL5-NM-002985.2-Reference-end	6.57000E-05	0.26947	1.74641E-02
	EGR1-NM-001964.2-Reference-end	8.00000E-05	0.43527	2.12748E-02
	CD300A-NM-007261.3-Reference-end	9.63000E-05	0.27694	2.56273E-02
	GZMH-NM-033423.4-Reference-end	1.19461E-04	0.40743	3.17766E-02
Cluster 9	CD38-CD38-AHS0022-pAbO	1.20795E-04	-0.36911	3.21314E-02
	HLA-DR-CD74-AHS0035-pAbO	5.14000E-06	-0.38355	1.36609E-03
	CD196-CCR6-AHS0034-pAbO	5.59000E-07	-0.45994	1.48753E-04
	CD3-CD3E-AHS0033-pAbO	1.44000E-08	-0.26143	3.84000E-06
	KLRC3-NM-002261.2-Reference-end	1.28000E-34	0.34221	3.40000E-32
	GZMK-NM-002104.2-Reference-end	8.07000E-24	0.29480	2.15000E-21
	TNF-NM-000594.3-Reference-end	3.68000E-18	0.43820	9.80000E-16
	TNFSF10-NM-003810.3-Reference-end	5.36000E-18	0.34028	1.43000E-15
	CD8B-NM-004931.4-Reference-end	2.84000E-15	0.39876	7.56000E-13
	STAT1-NM-007315.3-Reference-end	1.29000E-14	0.29101	3.43000E-12
	TIAF1-NM-004740.3-Reference-end	9.20000E-12	0.27440	2.45000E-09
	LEF1-NM-016269.4-Reference-end	9.46000E-10	0.34746	2.52000E-07
	CXCR3-NM-001504.1-Reference-end	1.53000E-09	0.25885	4.07000E-07
	EGR1-NM-001964.2-Reference-end	2.70000E-07	0.97612	7.19000E-05
	CD103-ITGAE-AHS0001-pAbO	1.38000E-06	0.59997	3.67739E-04
	CD8-CD8A-AHS0027-pAbO	4.15000E-06	0.71939	1.10388E-03
	LCK-NM-005356.4-Reference-end	6.16000E-06	0.39520	1.63919E-03
	CD69-NM-001781.2-Reference-end	1.09000E-05	0.46778	2.89853E-03
	HLA-DMA-NM-006120.3-Reference-end	1.48000E-05	0.27057	3.92572E-03
	CST7-NM-003650.3-Reference-end	7.22000E-05	0.32316	1.91999E-02
	LAG3-NM-002286.5-Reference-end	7.75000E-05	0.25950	2.06151E-02
	CD7-NM-006137.6-Reference-end	8.57000E-05	0.30481	2.27907E-02
Cluster 10	ITGAE-NM-002208.4-Reference-end	7.20000E-11	0.43849	1.92000E-08
	TNF-NM-000594.3-Reference-end	4.82000E-10	0.42350	1.28000E-07
	GIMAP2-NM-015660.2-Reference-end	6.13000E-09	0.27861	1.63000E-06
	ITK-NM-005546.3-Reference-end	1.00000E-08	0.38441	2.66000E-06
	ITGAM-NM-000632.3-Reference-end	7.85000E-08	0.40989	2.09000E-05
	RORA-NM-002943.3-Reference-end	1.26000E-07	0.41593	3.36000E-05
	NCR3-NM-147130.2-Reference-end	1.93000E-07	0.41372	5.15000E-05
	EGR1-NM-001964.2-Reference-end	2.47000E-07	0.54253	6.58000E-05
	TIGIT-ENST00000481065.5-Reference-en	2.63000E-07	0.34543	6.99000E-05
	IFNG-NM-000619.2-Reference-end	4.58000E-06	0.25913	1.21773E-03
	TSPAN32-NM-139022.2-Reference-end	2.03000E-05	0.26987	5.39576E-03
	HLA-DRA-NM-019111.4-Reference-end	3.97000E-05	0.55835	1.05712E-02
	CBLB-NM-170662.4-PolyA-1	5.28000E-05	0.61271	1.40419E-02
	FAS-NM-000043.5-PolyA-1	1.14786E-04	0.30248	3.05331E-02
	CD27-CD27-AHS0025-pAbO	4.10000E-05	-0.80823	1.09018E-02
	IL18-NM-001562.3-Reference-end	3.22000E-57	0.28327	8.57000E-55
	TRAT1-NM-016388.3-Reference-end	2.80000E-41	0.29560	7.44000E-39
	BCL2-NM-000633.2-Reference-end	2.08000E-33	0.36933	5.54000E-31

	GZMK-NM-002104.2-Reference-end	1.92000E-32	0.42508	5.11000E-30
	LAP3-NM-015907.2-Reference-end	1.38000E-31	0.32798	3.68000E-29
	IL18R1-NM-003855.3-Reference-end	6.28000E-30	0.33591	1.67000E-27
	HLA-DRB3-NM-022555.3-Reference-end	4.53000E-28	0.65202	1.21000E-25
	IL18RAP-NM-003853.3-Reference-end	3.41000E-25	0.42437	9.06000E-23
	STAT1-NM-007315.3-Reference-end	3.71000E-25	0.44591	9.87000E-23
	CD5-NM-014207.3-Reference-end	4.33000E-25	0.38385	1.15000E-22
	HLA-DMA-NM-006120.3-Reference-end	1.15000E-24	0.30089	3.06000E-22
	IL4R-NM-000418.3-Reference-end	5.42000E-22	0.35212	1.44000E-19
	ZNF683-NM-173574.3-Reference-end	5.42000E-22	0.74799	1.44000E-19
	KLRC1-NM-002259.4-Reference-end	8.55000E-22	0.52699	2.27000E-19
	IL12RB1-NM-005535.2-Reference-end	1.88000E-20	0.43935	5.00000E-18
	CLEC2D-NM-013269.5-PolyA-1	2.21000E-18	0.35413	5.87000E-16
	IL7R-NM-002185.3-Reference-end	1.14000E-17	0.53612	3.02000E-15
	CD160-NM-007053.3-Reference-end	1.62000E-14	0.66781	4.32000E-12
	LAT2-NM-014146.3-Reference-end	2.08000E-14	0.42064	5.53000E-12
	GZMM-NM-005317.3-Reference-end	3.37000E-11	0.33172	8.95000E-09
Cluster 11	ANXA5-NM-001154.3-Reference-end	1.71000E-28	0.28339	4.54000E-26
	CNOT2-NM-014515.5-Reference-end	7.97000E-28	0.32737	2.12000E-25
	TBX21-NM-013351.1-Reference-end	1.54000E-27	0.34369	4.10000E-25
	IL18R1-NM-003855.3-Reference-end	1.90000E-26	0.33910	5.06000E-24
	PRF1-NM-005041.4-Reference-end	2.11000E-25	0.35255	5.61000E-23
	CD300A-NM-007261.3-Reference-end	6.42000E-25	0.61241	1.71000E-22
	TRIB2-NM-021643.3-Reference-end	8.04000E-25	0.27512	2.14000E-22
	STAT3-NM-003150.3-Reference-end	1.07000E-24	0.49665	2.85000E-22
	ARL4C-NM-005737.3-Reference-end	2.42000E-23	0.25322	6.43000E-21
	TNF-NM-000594.3-Reference-end	7.79000E-23	0.51000	2.07000E-20
	TXK-NM-003328.2-Reference-end	4.02000E-22	0.48293	1.07000E-19
	IL4R-NM-000418.3-Reference-end	3.43000E-21	0.27712	9.12000E-19
	BCL11B-NM-022898.2-Reference-end	8.28000E-21	0.56685	2.20000E-18
	GZMB-NM-004131.4-Reference-end	3.54000E-20	0.37574	9.40000E-18
	CXCL8-NM-000584.3-Reference-end	8.36000E-20	0.40751	2.22000E-17
	TNFRSF1B-NM-001066.2-Reference-end	4.48000E-19	0.25978	1.19000E-16
	STAT6-NM-003153.4-Reference-end	1.86000E-17	0.37959	4.95000E-15
	TIAF1-NM-004740.3-Reference-end	3.66000E-17	0.43613	9.72000E-15
	SPOCK2-NM-014767.2-Reference-end	4.03000E-17	0.48163	1.07000E-14
	ZAP70-NM-001079.3-Reference-end	4.36000E-17	0.35896	1.16000E-14
	RORA-NM-002943.3-Reference-end	1.40000E-14	0.80919	3.72000E-12
	CTSW-NM-001335.3-Reference-end	1.62000E-13	0.34938	4.32000E-11
	FOSB-NM-006732.2-Reference-end	1.67000E-13	2.30178	4.44000E-11
	IL18RAP-NM-003853.3-Reference-end	1.93000E-11	0.38997	5.14000E-09
	JUN-NM-002228.3-Reference-end	5.07000E-11	0.53797	1.35000E-08
	ITGA4-NM-000885.5-PolyA-1	1.77000E-10	0.48842	4.70000E-08
	KLRK1-NM-007360.3-Reference-end	2.59000E-10	0.61798	6.89000E-08
	TARP-refseq-NM-001003799.1-Reference	7.30000E-10	0.34492	1.94000E-07
	ITK-NM-005546.3-Reference-end	7.58000E-10	0.57061	2.02000E-07
	BIN2-NM-016293.3-Reference-end	1.42000E-09	0.35821	3.79000E-07
	IER5-NM-016545.4-Reference-end	3.84000E-08	0.33531	1.02000E-05
	LEF1-NM-016269.4-Reference-end	1.12000E-07	0.63095	2.99000E-05
	FYN-NM-002037.5-PolyA-1	2.22000E-07	0.37863	5.89000E-05
	CBLB-NM-170662.4-PolyA-1	5.43000E-07	0.75550	1.44492E-04
	GNLY-NM-006433.4-Reference-end	8.29000E-07	0.60458	2.20442E-04
	CD6-NM-006725.4-Reference-end	1.93000E-06	0.49113	5.13912E-04
	FYB-NM-001465.4-Reference-end	2.89000E-06	0.53783	7.69407E-04
	IL23R-NM-144701.2-Reference-end	9.03000E-06	0.93166	2.40284E-03
	CD44-CD44-AHS0054-pAbO	1.39000E-05	0.26812	3.69676E-03
	LCK-NM-005356.4-Reference-end	1.75000E-05	0.36491	4.64925E-03
	NKG7-NM-005601.3-Reference-end	4.29000E-05	0.29206	1.14161E-02
	HLA-DR-CD74-AHS0035-pAbO	1.84299E-04	-0.90662	4.90234E-02
	GAPDH-NM-002046.5-Reference-end	8.31000E-09	-0.28805	2.21000E-06
	IL2RB-NM-000878.3-Reference-end	5.01000E-29	-0.26116	1.33000E-26
	FOXO1-NM-002015.3-Reference-end	1.87000E-48	0.29349	4.97000E-46
	CLEC2D-NM-013269.5-PolyA-1	9.64000E-39	0.26137	2.56000E-36
	IL18-NM-001562.3-Reference-end	1.45000E-37	0.41002	3.86000E-35
	IFNG-NM-000619.2-Reference-end	2.29000E-36	0.35120	6.09000E-34
	KLRG1-NM-005810.3-Reference-end	2.26000E-35	0.50335	6.02000E-33
	STAT4-NM-003151.3-Reference-end	2.82000E-35	0.34017	7.49000E-33
	NAMPT-NM-005746.2-PolyA-1	1.94000E-32	0.58635	5.17000E-30
	IFNGR1-NM-000416.2-Reference-end	9.98000E-31	0.31265	2.66000E-28
	ANXA5-NM-001154.3-Reference-end	1.71000E-28	0.28339	4.54000E-26

	CNOT2-NM-014515.5-Reference-end	7.97000E-28	0.32737	2.12000E-25
	TBX21-NM-013351.1-Reference-end	1.54000E-27	0.34369	4.10000E-25
	IL18R1-NM-003855.3-Reference-end	1.90000E-26	0.33910	5.06000E-24
	PRF1-NM-005041.4-Reference-end	2.11000E-25	0.35255	5.61000E-23
	CD300A-NM-007261.3-Reference-end	6.42000E-25	0.61241	1.71000E-22
	TRIB2-NM-021643.3-Reference-end	8.04000E-25	0.27512	2.14000E-22
	STAT3-NM-003150.3-Reference-end	1.07000E-24	0.49665	2.85000E-22
	ARL4C-NM-005737.3-Reference-end	2.42000E-23	0.25322	6.43000E-21
	TNF-NM-000594.3-Reference-end	7.79000E-23	0.51000	2.07000E-20
	TXK-NM-003328.2-Reference-end	4.02000E-22	0.48293	1.07000E-19
	IL4R-NM-000418.3-Reference-end	3.43000E-21	0.27712	9.12000E-19
Cluster 12	CXCL8-NM-000584.3-Reference-end	7.07000E-39	0.30834	1.88000E-36
	TRDC-ENST00000390477.2-Reference-er	2.90000E-11	0.62531	7.72000E-09
	DUSP4-NM-001394.6-Reference-end	6.87000E-07	0.50422	1.82825E-04
	CD300A-NM-007261.3-Reference-end	1.11000E-06	0.32512	2.95131E-04
	LGALS1-NM-002305.3-Reference-end	1.13000E-06	1.05130	3.00903E-04
	LGALS3-NM-002306.3-Reference-end	1.72000E-06	0.41942	4.57531E-04
	ANXA5-NM-001154.3-Reference-end	2.42000E-06	0.83047	6.42502E-04
	CD3D-NM-000732.4-Reference-end	4.94000E-06	0.44117	1.31521E-03
	STAT4-NM-003151.3-Reference-end	5.01000E-06	0.25966	1.33218E-03
	ZNF683-NM-173574.3-Reference-end	6.27000E-06	0.28776	1.66913E-03
	CCR7-NM-001838.3-Reference-end	5.50000E-05	0.27688	1.46392E-02
	GAPDH-NM-002046.5-Reference-end	7.85000E-05	0.76121	2.08685E-02
	AURKB-NM-004217.3-Reference-end	1.24897E-04	0.51956	3.32225E-02
	FOXO3-NM-001455.3-Reference-end	1.42000E-04	0.26038	3.77721E-02
	CD27-CD27-AHS0025-pAbO	3.38000E-06	-0.48871	8.99433E-04
	CXCL8-NM-000584.3-Reference-end	7.07000E-39	0.30834	1.88000E-36
	TRDC-ENST00000390477.2-Reference-er	2.90000E-11	0.62531	7.72000E-09
	DUSP4-NM-001394.6-Reference-end	6.87000E-07	0.50422	1.82825E-04
	CD300A-NM-007261.3-Reference-end	1.11000E-06	0.32512	2.95131E-04
	LGALS1-NM-002305.3-Reference-end	1.13000E-06	1.05130	3.00903E-04
	LGALS3-NM-002306.3-Reference-end	1.72000E-06	0.41942	4.57531E-04
	ANXA5-NM-001154.3-Reference-end	2.42000E-06	0.83047	6.42502E-04
	CD3D-NM-000732.4-Reference-end	4.94000E-06	0.44117	1.31521E-03
	STAT4-NM-003151.3-Reference-end	5.01000E-06	0.25966	1.33218E-03
	ZNF683-NM-173574.3-Reference-end	6.27000E-06	0.28776	1.66913E-03
	CCR7-NM-001838.3-Reference-end	5.50000E-05	0.27688	1.46392E-02
	GAPDH-NM-002046.5-Reference-end	7.85000E-05	0.76121	2.08685E-02
	AURKB-NM-004217.3-Reference-end	1.24897E-04	0.51956	3.32225E-02
	FOXO3-NM-001455.3-Reference-end	1.42000E-04	0.26038	3.77721E-02
Cluster 13	PIK3IP1-NM-052880.4-Reference-end	7.89000E-08	0.58602	2.10000E-05
	IFNG-NM-000619.2-Reference-end	1.08000E-06	0.26419	2.87763E-04
	TRAC-ENST00000611116.1-Reference-er	1.68000E-06	0.84242	4.46406E-04
	ZNF683-NM-173574.3-Reference-end	1.93000E-06	0.44193	5.12685E-04
	CCR7-NM-001838.3-Reference-end	8.20000E-06	0.26144	2.18004E-03
	CXCR4-NM-003467.2-Reference-end	1.28000E-05	0.52090	3.41119E-03
	IL32-NM-004221.4-Reference-end	1.78000E-05	0.65136	4.73122E-03
	CD3E-NM-000733.3-Reference-end	4.55000E-05	0.56890	1.21076E-02
	CD3-CD3E-AHS0033-pAbO	7.34000E-05	0.49326	1.95222E-02
	TRBC2-ENST00000466254.1-Reference-e	1.16791E-04	0.66359	3.10663E-02
	RUNX3-NM-004350.2-Reference-end	1.45284E-04	0.66574	3.86455E-02
Cluster 14	LGALS3-NM-002306.3-Reference-end	3.26922E-24	-0.81812	8.69613E-22
	IL6R-NM-000565.3-Reference-end	1.02340E-15	0.31737	2.72223E-13
	IER3-NM-003897.3-Reference-end	5.78150E-14	0.79192	1.53788E-11
	CCL3-NM-002983.2-Reference-end	6.31930E-14	0.27328	1.68093E-11
	TRDC-ENST00000390477.2-Reference-er	1.15324E-13	0.64660	3.06762E-11
	PASK-NM-015148.3-Reference-end	2.00694E-13	0.27535	5.33846E-11
	KLRF1-NM-016523.2-Reference-end	1.22698E-12	0.47898	3.26378E-10
	LAP3-NM-015907.2-Reference-end	3.08732E-12	0.25229	8.21226E-10
	CCR3-NM-001837.3-Reference-end	3.58662E-11	-0.27209	9.54040E-09
	LAIR2-NM-002288.5-Reference-end	7.98826E-10	-0.43466	2.12488E-07
	ZAP70-NM-001079.3-Reference-end	3.75776E-09	0.25671	9.99565E-07
	CD7-NM-006137.6-Reference-end	1.17379E-07	0.43533	3.12228E-05
	CST7-NM-003650.3-Reference-end	1.56272E-07	1.10992	4.15685E-05
	ITK-NM-005546.3-Reference-end	4.06776E-07	0.55969	1.08202E-04
	ZNF683-NM-173574.3-Reference-end	5.13091E-07	0.40471	1.36482E-04
	KLRG1-NM-005810.3-Reference-end	6.67501E-07	0.40631	1.77555E-04
	TRIB2-NM-021643.3-Reference-end	2.10442E-06	0.34836	5.59776E-04
	HMGB2-NM-002129.3-PolyA-1	3.71144E-06	0.31546	9.87242E-04
	HLA-DRA-NM-019111.4-Reference-end	5.44263E-06	0.49142	1.44774E-03

FOXO3-NM-001455.3-Reference-end	7.91027E-06	0.27022	2.10413E-03
TBX21-NM-013351.1-Reference-end	9.18509E-06	0.37513	2.44323E-03
CCL4-NM-002984.3-Reference-end	4.56448E-05	0.56170	1.21415E-02
CNOT2-NM-014515.5-Reference-end	7.05796E-05	0.28666	1.87742E-02
LGALS1-NM-002305.3-Reference-end	8.70892E-05	0.65088	2.31657E-02
CD4-NM-000616.4-Reference-end	8.81801E-05	0.40481	2.34559E-02
APOBEC3G-NM-021822.3-Reference-end	1.82377E-04	0.41541	4.85124E-02
