# Supplementary material for: Single‐cell multi‐omics analysis presents the landscape of peripheral blood T‐cell subsets in human chronic prostatitis/chronic pelvic pain syndrome
Source: J Cell Mol Med. 2020 Oct 30;24(23):14099–109. doi: 10.1111/jcmm.16021 (PMC7754003; doi:10.1111/jcmm.16021)
Supplement: Supplementary file 17 — Table S7 [file JCMM-24-14099-s017.pdf]

Supplementary table 7. The differentially expressed genes between CP/CPPS and healthy controls

Gene_ID	*P*_value	Average_logFoldChange	*P*_value_adjusted
CD244-NM-016382.3	0.0000	0.2513	0.0000
LAG3-NM-002286.5	0.0000	0.2528	0.0000
HLA-DRB3-NM-022555.3	0.0000	0.3473	0.0000
TRDC-ENST00000390477.2	0.0000	0.4956	0.0000
LAIR2-NM-002288.5	0.0000	0.2844	0.0000
ITGAM-NM-000632.3	0.0000	0.2604	0.0000
TNF-NM-000594.3	0.0000	0.3834	0.0000
IFNG-NM-000619.2	0.0000	0.3606	0.0000
ZNF683-NM-173574.3	0.0000	0.3364	0.0000
GZMK-NM-002104.2	0.0000	0.2683	0.0000
CCL4-NM-002984.3	0.0000	0.3597	0.0000
CBLB-NM-170662.4-PolyA-1	0.0000	0.3307	0.0000
CTSW-NM-001335.3	0.0000	0.4130	0.0000
LCK-NM-005356.4	0.0000	0.2582	0.0000
ITGAL-NM-002209.2	0.0000	0.2965	0.0000
BIN2-NM-016293.3	0.0000	0.2621	0.0000
CCL5-NM-002985.2	0.0000	0.3407	0.0000
NKG7-NM-005601.3	0.0000	0.3968	0.0000
FYN-NM-002037.5-PolyA-1	0.0000	0.3557	0.0000
RUNX3-NM-004350.2	0.0000	0.3233	0.0000
CD2-NM-001767.3	0.0000	0.2656	0.0000
IER5-NM-016545.4	0.0000	0.3194	0.0000
BCL11B-NM-022898.2	0.0000	0.2541	0.0000
CST7-NM-003650.3	0.0000	0.3911	0.0000
TNFRSF1B-NM-001066.2	0.0000	0.2948	0.0000
TARP-refseq-NM-00100379	0.0000	0.4233	0.0000
KLRG1-NM-005810.3	0.0000	0.3483	0.0000
FOSB-NM-006732.2	0.0000	0.3697	0.0000
TRIB2-NM-021643.3	0.0000	0.2814	0.0000
FYB-NM-001465.4	0.0000	0.3225	0.0000
HLA-DPB1-NM-002121.5-Po	0.0000	0.3592	0.0000
SELPLG-NM-003006.4-Poly	0.0000	0.2840	0.0000
CD300A-NM-007261.3	0.0000	0.4119	0.0000
LGALS1-NM-002305.3	0.0000	0.4232	0.0000
DUSP2-NM-004418.3	0.0000	0.3462	0.0000
JUN-NM-002228.3	0.0000	0.3531	0.0000
TBX21-NM-013351.1	0.0000	0.3968	0.0000
CD8A-NM-001768.6	0.0000	0.3619	0.0000
ARL4C-NM-005737.3	0.0000	0.2830	0.0000
GZMH-NM-033423.4	0.0000	0.3858	0.0000
GZMA-NM-006144.3	0.0000	0.3079	0.0000
KLRK1-NM-007360.3	0.0000	0.3185	0.0000
GNLY-NM-006433.4	0.0000	0.3847	0.0000
KLRB1-NM-002258.2	0.0000	0.3826	0.0000
ANXA5-NM-001154.3	0.0000	0.3179	0.0000
BAX-NM-001291428.1	0.0000	0.3203	0.0002
IL2RB-NM-000878.3	0.0000	0.3237	0.0007
APOBEC3G-NM-021822.3	0.0001	0.3720	0.0149
CD247-NM-000734.3	0.0001	0.2928	0.0173
SPOCK2-NM-014767.2	0.0001	0.2922	0.0344
ITGA4-NM-000885.5-PolyA-	0.0016	0.3609	0.4351
GZMB-NM-004131.4	0.0017	0.4354	0.4640
EGR1-NM-001964.2	0.0023	0.7536	0.6015
HLA-DPA1-NM-033554.3-Po	0.0243	0.3548	1.0000
PRF1-NM-005041.4	0.0843	0.3949	1.0000
CD5-NM-014207.3	0.1464	0.2790	1.0000

CP/CPPS, chronic prostatitis/chronic pelvic pain syndrome
