# Supplementary material for: Single‐cell multi‐omics analysis presents the landscape of peripheral blood T‐cell subsets in human chronic prostatitis/chronic pelvic pain syndrome
Source: J Cell Mol Med. 2020 Oct 30;24(23):14099–109. doi: 10.1111/jcmm.16021 (PMC7754003; doi:10.1111/jcmm.16021)
Supplement: Supplementary file 18 — Table S8 [file JCMM-24-14099-s018.pdf]

**Supplementary table 8.** Differentially expressed genes between cells derived from prostatitis patients and healthy controls in each cluster.

| Cluster ID | Gene symbol | *P* _value | Average_logFoldChange | *P* _value_adjusted |
|---|---|---|---|---|
| Cluster 0 | CD27-NM-001242.4-Reference-end | 1.55336E-12 | 0.31431 | 4.13194E-10 |
| | GZMB-NM-004131.4-Reference-end | 5.10513E-11 | 0.35931 | 1.35796E-08 |
| | GIMAP2-NM-015660.2-Reference-end | 3.58139E-09 | 0.25652 | 9.52649E-07 |
| | LGALS1-NM-002305.3-Reference-end | 8.91670E-08 | 0.25440 | 2.37184E-05 |
| | CD103-ITGAE-AHS0001-pAbO | 2.43261E-06 | 0.56601 | 6.47074E-04 |
| | CD8B-NM-004931.4-Reference-end | 8.45332E-06 | 0.28647 | 2.24858E-03 |
| Cluster 1 | DPP4-NM-001935.3-Reference-end | 4.42000E-11 | 0.26495 | 1.18000E-08 |
| | LTA-NM-000595.3-Reference-end | 3.72000E-10 | 0.27332 | 9.89000E-08 |
| | TXK-NM-003328.2-Reference-end | 7.68000E-07 | 0.36320 | 2.04223E-04 |
| | CTSW-NM-001335.3-Reference-end | 1.69731E-04 | 0.41515 | 4.51486E-02 |
| Cluster 2 | LGALS1-NM-002305.3-Reference-end | 8.67633E-06 | 0.43251 | 2.30790E-03 |
| | CCR7-NM-001838.3-Reference-end | 1.99883E-05 | 0.25967 | 5.31689E-03 |
| Cluster 3 | IL7R-NM-002185.3-Reference-end | 1.48584E-08 | 0.41380 | 3.95234E-06 |
| | HLA-DR-CD74-AHS0035-pAbO | 2.79495E-06 | -1.11451 | 7.43455E-04 |
| | CTSW-NM-001335.3-Reference-end | 3.69445E-06 | 0.33373 | 9.82724E-04 |
| | PIK3IP1-NM-052880.4-Reference-end | 7.44565E-06 | 0.27633 | 1.98054E-03 |
| | SELL-NM-000655.4-Reference-end | 1.43489E-05 | 0.31352 | 3.81680E-03 |
| | IER5-NM-016545.4-Reference-end | 5.35928E-05 | 0.25910 | 1.42557E-02 |
| | CD45RO-PTPRC-AHS0036-pAbO | 1.75104E-04 | -0.25176 | 4.65776E-02 |
| Cluster 4 | CD27-NM-001242.4-Reference-end | 2.09000E-07 | 0.59487 | 5.55000E-05 |
| | BCL2-NM-000633.2-Reference-end | 6.56000E-07 | 0.32652 | 1.74592E-04 |
| | TXK-NM-003328.2-Reference-end | 5.79000E-06 | 0.32596 | 1.53898E-03 |
| | LCK-NM-005356.4-Reference-end | 9.39000E-06 | 0.35440 | 2.49667E-03 |
| | PIK3IP1-NM-052880.4-Reference-end | 1.02000E-05 | 0.43209 | 2.70189E-03 |
| | FYB-NM-001465.4-Reference-end | 1.54000E-05 | 0.41301 | 4.09430E-03 |
| | CTLA4-NM-005214.4-Reference-end | 3.04000E-05 | 0.25163 | 8.07845E-03 |
| | CD2-NM-001767.3-Reference-end | 6.05000E-05 | 0.41520 | 1.60983E-02 |
| | LEF1-NM-016269.4-Reference-end | 8.78000E-05 | 0.56147 | 2.33490E-02 |
| | CD5-NM-014207.3-Reference-end | 1.35745E-04 | 0.50933 | 3.61081E-02 |
| | SELL-NM-000655.4-Reference-end | 1.80873E-04 | 0.35532 | 4.81121E-02 |

**Note:** There was no significant differentially expressed genes between cells derived from prostatitis patients and healthy controls in cluster 5.
