# Supplementary material for: Single‐cell multi‐omics analysis presents the landscape of peripheral blood T‐cell subsets in human chronic prostatitis/chronic pelvic pain syndrome
Source: J Cell Mol Med. 2020 Oct 30;24(23):14099–109. doi: 10.1111/jcmm.16021 (PMC7754003; doi:10.1111/jcmm.16021)
Supplement: Supplementary file 2 — Fig S2 [file JCMM-24-14099-s002.pdf]

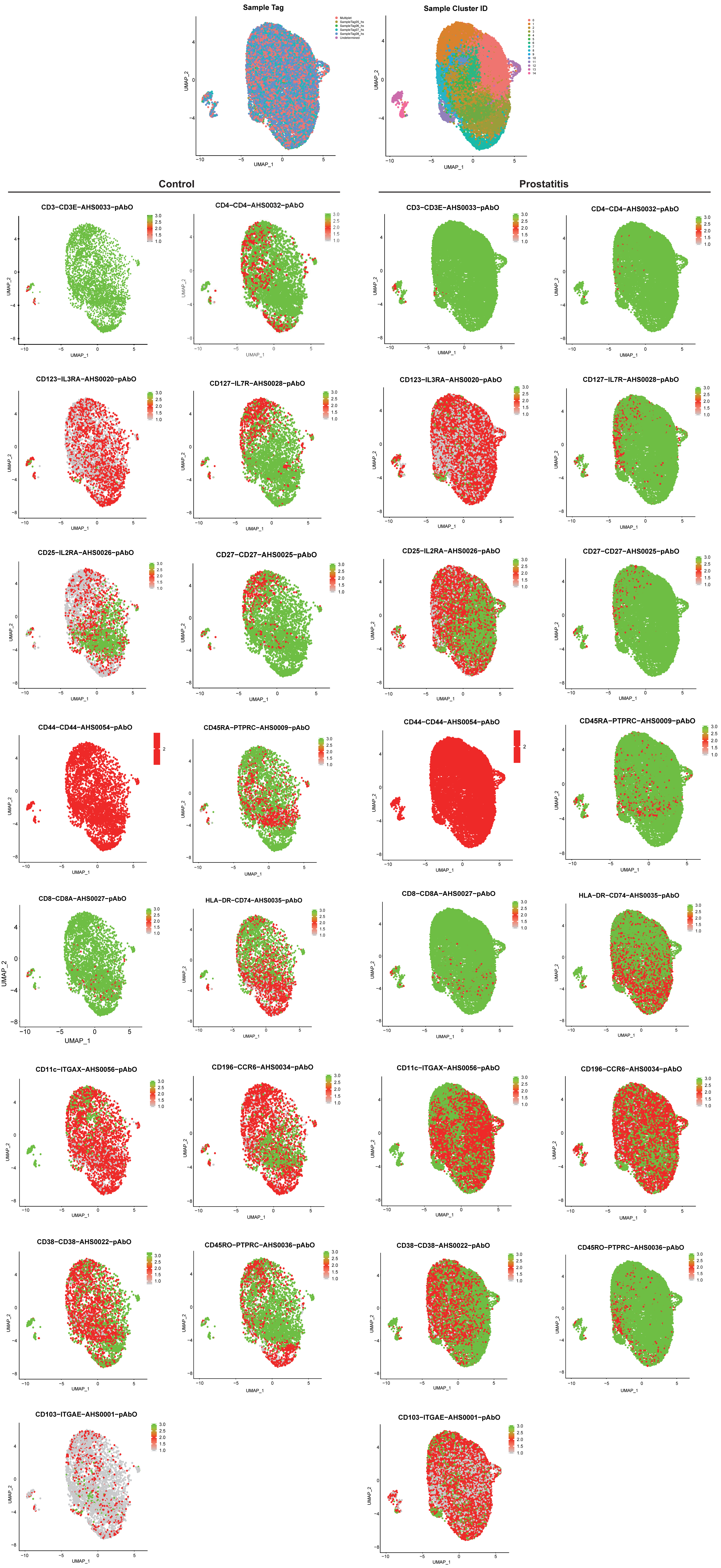

Supplementary figure 2. Protein abundance of the fifteen antibody-tagged genes in the control group (A) and case group (B). Note: Tag05_hs & Tag06_hs were healthy controls, while Tag7 & Tag8 were chronic prostatitis cases.
